# Disentangling biotic and abiotic drivers of a neotropical mistletoe

**DOI:** 10.1007/s00442-026-05975-6

**Published:** 2026-09-15

**Authors:** Eduardo V. S. Oliveira, Thieres Santos Almeida, Helon Simões Oliveira, Sidney F. Gouveia

**Affiliations:** 1 Department of Ecology, Federal University of Sergipe, São Cristóvão, Sergipe Brazil; 2 Graduate Program in Ecology and Conservation, Federal University of Sergipe, São Cristóvão, Sergipe Brazil

**Keywords:** *Psittacanthus robustus*, Niche overlap, Vochysiaceae, *Tersina viridis viridis*, *Elaenia cristata*, Shapley Additive Explanation values

## Abstract

Parasitic plants known as mistletoes are a key resource in ecosystems and form intricate networks of biotic interactions. Several mistletoe species exhibit specific associations with particular hosts and dispersers, such as *Psittacanthus robustus*, which is distributed across South America. Here, we assessed the climatic niche similarity between *P. robustus*, its host trees, and seed dispersers, and identified the main factors influencing the parasite’s occurrence. We analyzed the spatial overlap among *P. robustus*, hosts, and seed dispersers using niche equivalence and similarity tests and assessed the importance of predictors through machine-learning classification. We detected higher climatic niche similarity between *P. robustus* and its host trees, while niche equivalence was absent in all comparisons. The spatial distribution of *P. robustus* was primarily shaped by the presence of host trees rather than climatic conditions or seed dispersers. These results highlight the impact of host trees on the spatial distribution of *P. robustus*, serving as ecological filters that influence mistletoe occurrence. The secondary role of climate and dispersers likely arises from interacting processes such as climatic predictability, mistletoe reproductive phenology, host vulnerability, and the asymmetric dependence of mutualist animals on mistletoe resources.

**Supplementary Information:**

The online version contains supplementary material available at https://doi.org/10.1007/s00442-026-05975-6.

## Introduction

Species distributions are related to different drivers, including evolutionary history, abiotic factors, and biotic interactions (Gutiérrez et al. 2014). Abiotic factors are usually considered key determinants of species distributions on macroecological scales, whereas biotic interactions are frequently presumed to influence species distributions on local scales (Wisz et al. 2013; Cosentino et al. 2023). Therefore, in most studies, biotic interactions are not integrated into the analysis of species’ range limits (Giannini et al. 2013; Anderson 2017). In recent years, efforts have yielded new evidence to comprehend the influence of biotic interactions on species distribution at large scales (Godsoe et al. 2017; Dormann et al. 2018; Paquette and Hargreaves 2021). In addition to the debate on whether biotic interactions result in geographical dependence at macroecological scales (Araujo and Rozenfeld 2014), there has also been discussion about the signature of different types of biotic interactions, such as competition, predation, parasitism, and mutualism, in affecting species distributions on broad scales (Wisz et al. 2013).

Specifically, two types of biotic interactions, namely host-parasite and mutualism, may play an important role in shaping the distribution of species at large scales (Giannini et al. 2013; Wisz et al. 2013). The higher effect of host species’ presence than abiotic factors on the prevalence and distribution of parasites has been documented for different groups (e.g., Fecchio et al. 2018; Gustafson et al. 2018; Ellis et al. 2020; Rodríguez et al. 2022). However, there is also evidence of the combined effect of host species’ presence and climatic factors on parasite distributions (e.g., Padilla et al. 2017; Clark et al. 2018; Fecchio et al. 2019). Similarly, positive interactions, such as mutualism, have the potential to modify species distribution (Vasconcelos et al. 2017; Narayanan and Shaw 2024). This is the case with angiosperms’ dependence on specific animals for seed dispersal (Rogers et al. 2021). The effects of seed dispersal have a marked influence on the distribution of plants and are among the most important predictors that control the geographic occurrence of angiosperms (Beckman and Sullivan 2023; Marchioro et al. 2023).

Mistletoes are hemiparasitic plants involved in an intricate network of biotic interactions, including parasitized hosts and mutualistic relationships with seed-dispersing animals (Watson 2001; Aukema 2003). A relevant question about the mistletoes is to understand the importance of abiotic factors and biotic interactions in their distribution at macroecological scales. It seems that the range of mistletoe species is influenced mainly by the availability of hosts (e.g., Sayad et al. 2017; Sasal et al. 2021), followed by climatic conditions (Ojeda et al. 2023; Wilson and Trang 2025) and seed dispersers (e.g., Martinez del Rio et al. 1996; Fadini et al. 2010; Okubamichael et al. 2016). Considering that the mistletoe’s ecological niche is restricted by the combination of climatic conditions and biotic interactions (Rohde 1994; Ramírez-Barahona et al. 2017), its distribution is expected to occur where suitable climatic conditions overlap with the presence of hosts and seed dispersers (Lira-Noriega and Peterson 2014; Lira-Noriega et al. 2015). This pattern is consistent with the ecology of specialized parasitic plants, in which strong associations with particular host species and dispersal agents can contribute to restricting the realized ecological niche (Okubamichael et al. 2016; Muche et al. 2022).

Most mistletoe species belong to the Loranthaceae family (order Santalales) (Lüttge et al. 1998). Within this family, *Psittacanthus robustus* (Mart.) Mart. (Loranthaceae) stands out as one of the most widespread species of its genus in Brazil (Kuijt 2009; Oliveira and Caires 2018). The species preferentially infects individuals of the Vochysiaceae family in open areas (savannas) of South America, having a notable affinity with the *Vochysia* and *Qualea* genera (Monteiro et al. 1992; Teodoro et al. 2010). Much of the seed dispersal service of *P. robustus* has been attributed to *Tersina viridis viridis* Illiger, 1811 (Thraupidae) (Monteiro et al. 1992). However, Guerra (2010) subsequently identified *Elaenia cristata* Pelzeln, 1868 (Tyrannidae) as the dominant seed disperser at another study site, whereas *T. viridis viridis* was only rarely observed. Both bird species consume the fruits of *P. robustus* and effectively disperse its seeds by swallowing whole fruits and subsequently regurgitating intact seeds onto host branches during bill-wiping behavior (Monteiro et al. 1992; Guerra 2010).

To date, few studies have examined how biotic interactions shape species distributions at macro-spatial scales in the tropics (Cosentino et al. 2023). The mistletoe *P. robustus* offers an ideal model for such studies because of its pronounced ecological associations and broad distribution. Using occurrence records and environmental predictors, we tested whether the identity of local interaction partners is reflected in the spatial and climatic niche similarity between *P. robustus* and its host plants and seed dispersers across South America. We expected to find (i) a similar climatic niche between the species, particularly between *P. robustus* and host trees, but without niche equivalence, and (ii) a stronger effect of hosts on the occurrence of *P. robustus* than that of seed-dispersing birds and climatic variables. This expectation is based on evidence that infection occurs under climatic conditions similar to those of the host, but within a portion of the host’s climate niche (Lira-Noriega and Peterson 2014). In addition, seed-dispersing birds generally have broader geographic distributions than *P. robustus* and therefore a wider range of environmental tolerance (Rubim 2009), whereas *P. robustus* shows a high dependence on a subset of host species (Wang et al. 2023; Zhao et al. 2023). Moreover, seed-dispersing birds exhibit an asymmetric dependence on mistletoes and are generally more opportunistic in their use of food resources (Guerra and Pizo 2014), leading us to expect a weaker contribution of dispersers than hosts to the spatial occurrence of *P. robustus*.

## Materials and methods

### Selection of the hosts and seed-disperser species

We defined the species of host plants and seed-dispersing birds of *Psittacanthus robustus* based on a bibliographic review. Seeking to guarantee precision in the analysis, we included only species with parasitism documented and birds explicitly reported as dispersing the seeds of *P. robustus* (Online Resource 1, Text S1). We defined 17 species of host trees: *Coccoloba cereifera* Schwacke, *Campomanesia adamantium* (Cambess.) O.Berg, *Diplusodon hirsutus* (Cham. & Schltdl.) A.DC., *Miconia albicans* (Sw.) Steud., *Miconia ferruginata* DC., *Qualea cordata* Spreng., *Qualea dichotoma* (Mart.) Warm., *Qualea grandiflora* Mart., *Qualea multiflora* Mart., *Qualea parviflora* Mart., *Trembleya laniflora* (D.Don) Cogn., *Salvertia convallariodora* A.St.-Hil., *Vochysia cinnamomea* Pohl, *Vochysia elliptica* Mart., *Vochysia rufa* Mart., *Vochysia tucanorum* Mart., and *Vochysia thyrsoidea* Pohl. Furthermore, we defined four seed-dispersing bird species: *Tersina viridis viridis* (Illiger, 1811), *Elaenia cristata* Pelzeln, 1868, *Thraupis sayaca sayaca* (Linnaeus, 1766), and *Schistochlamys ruficapillus* (Vieillot, 1817) (Online Resource 1, Table S1).

## Occurrence data and environmental layers

**Fig. 1:**
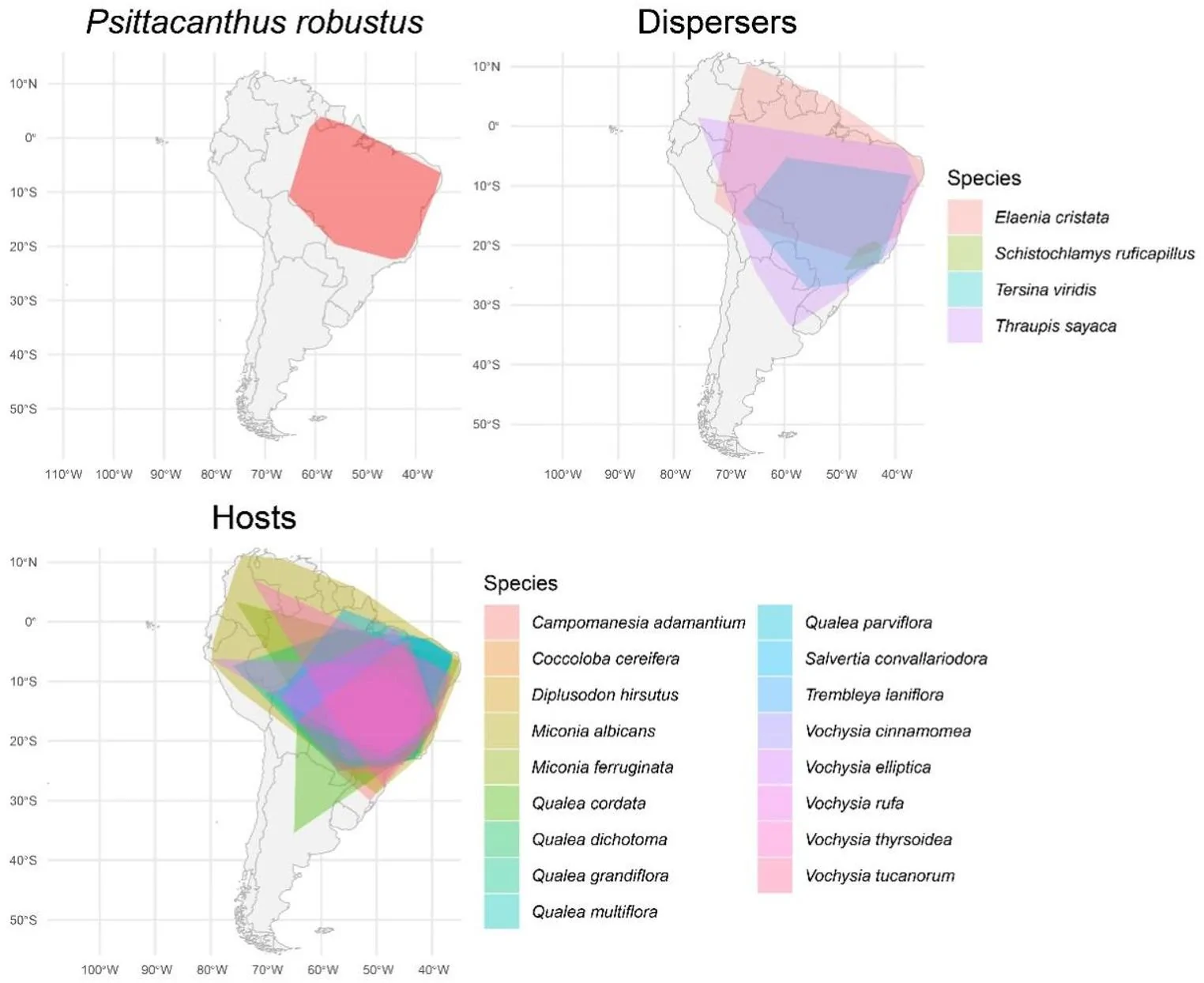
Convex hulls representing the geographic ranges of *Psittacanthus robustu*s, seed-dispersing birds, and host trees in South America, based on occurrence records

Occurrence data for *Psittacanthus robustus* and its host trees were obtained from the Botanical Information and Ecology Network – BIEN (https://bien.nceas.ucsb.edu/bien/). This database integrates and standardizes occurrence records of plants, providing clean data for analysis (Maitner et al. 2017). Bird occurrence data were gathered from the Global Biodiversity Information Facility – GBIF (https://www.gbif.org/) and speciesLink (https://specieslink.net). We complemented occurrence records of plants with these two latter databases. Species occurrence records were accessed from the R packages ‘BIEN’ (Maitner et al. 2017), ‘rgbif’ (https://cran.r-project.org/web/packages/rgbif), and ‘plantR’ (Lima et al., 2023). For the data of GBIF and speciesLink, we used a standardized cleaning procedure to remove invalid or duplicated coordinates using the R package ‘CoordinateCleaner‘ (Zizka et al. 2019). Then, we visually inspected the occurrence records and created convex hulls for each species (Fig. 1). After this step, we excluded the *Coccoloba cereifera* species from the analysis due to the insufficient number of occurrence records.

We preselected 19 environmental layers derived from temperature and precipitation data available in WorldClim (Fick and Hijmans 2017; Online Resource 1, Table S2). We eliminated multicollinearity by retaining only variables without a Pearson’s correlation coefficient greater than 0.7. Subsequently, we selected the mean diurnal range, isothermality, mean temperature of warmest quarter, precipitation of the wettest month, precipitation seasonality, precipitation of the warmest quarter, and precipitation of the coldest quarter for the analyses. Multicollinearity was detected and removed using the ‘virtualspecies’ R package (Leroy et al. 2016).

## Environmental overlap analysis

**Fig. 2:**
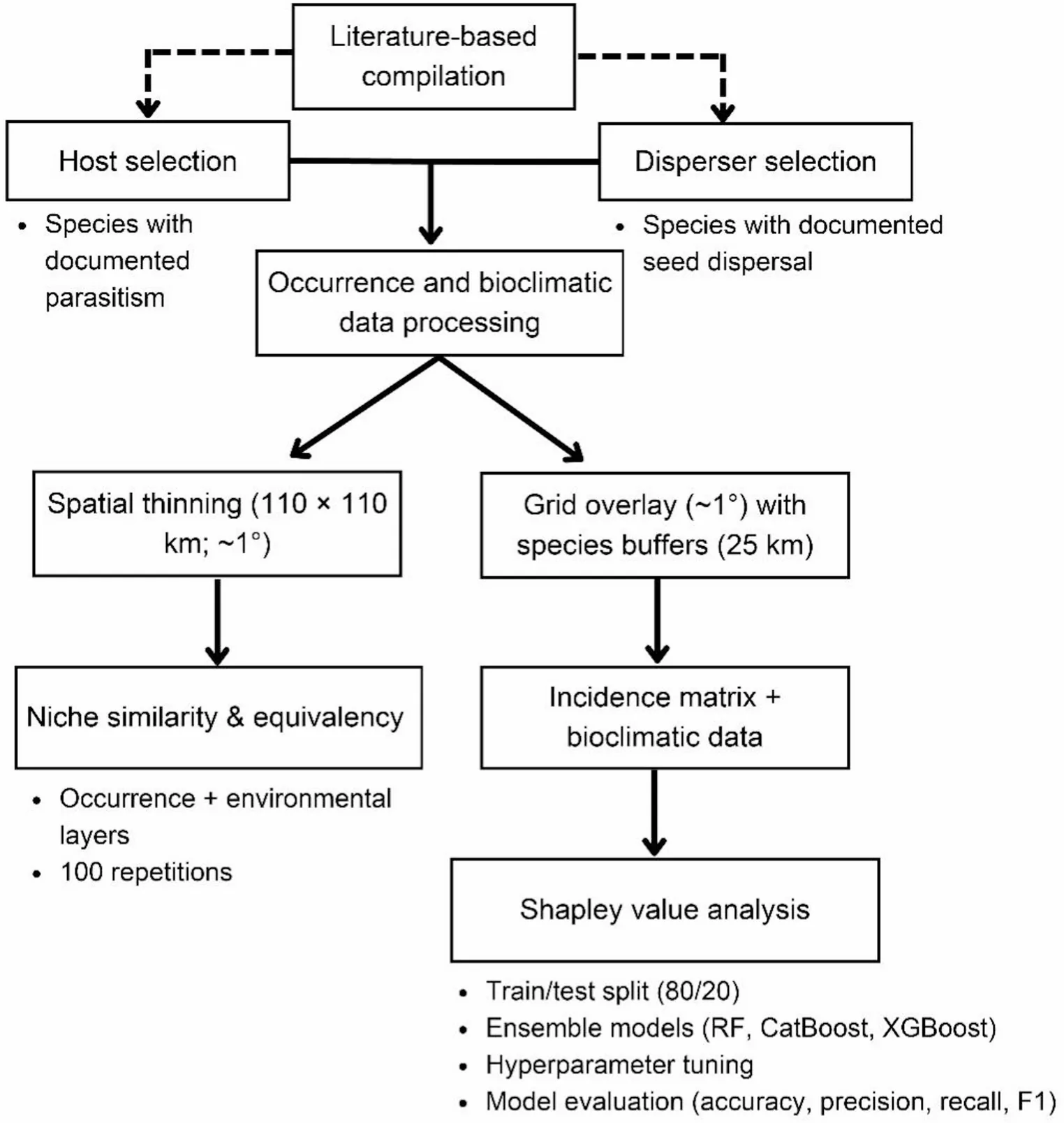
Flowchart illustrating the workflow used to compile data on host trees and seed-dispersing birds of *Psittacanthus robustus*, followed by data processing, niche overlap analyses, and Shapley value analysis

We estimated the overlap in the environmental space from the species occurrence density in the grid cells (110 × 110 km; ~1°) constructed around the occurrence, combined with the environmental layers (Fig. 2). Then, we calculated Schoener’s D overlap index, which informs an absence of overlap (zero) to an identical distribution (= 1) between the two species (Warren et al. 2008; Rodder and Engler 2011). Based on the overlap in environmental space, we performed niche equivalence and similarity tests with 100 repetitions (Warren et al. 2008). While the equivalence test evaluates whether the niches of two species are identical, the similarity test evaluates whether the niche of one species can predict the niche of another species (Aguirre-Gutiérrez et al. 2015). For these analyses, we used the ‘dismo’ (https://cran.r-project.org/web/packages/dismo) and ‘ecospat’ R packages (Cola et al. 2017).

## Machine learning and shapley values

From the occurrence records of species (*P. robustus*, hosts, and seed-disperser birds), we generated collapsed buffers with a 25 km radius. Then, we overlapped these buffers with grid cells (110 × 110 km; ~1°) of South America to create an incidence matrix for *P. robustus* (Online Resource 1, Table S3). Due to the higher number of cells (henceforth sites) with absences of *P. robustus*, unbalancing the dataset, we selected the same number of sites with their presence (i.e., 134) from a randomization procedure. Sites where absences are noted might still host species, including hosts and/or dispersers, or they might be devoid of species. Similarly, locations with *P*. *robustus* present could also have hosts and/or dispersers, or they might lack any species entirely. From the incidence matrix, we extracted the values of the seven climatic environmental layers independently described previously for each site. The final dataset used for the analysis comprised the presence–absence of the mistletoe *Psittacanthus robustus* (response variable), biotic predictors (presence–absence of host trees and seed-disperser birds), and abiotic predictors (7 climatic variables) (Table 1; Fig. 2). For these procedures, we used the ‘terra’ (https://cran.r-project.org/web/packages/terra) and ‘letsR’ R packages (Vilela and Villalobos 2015).

**Table 1:** Predictors used in machine learning models to classify the presence-absence of the mistletoe *Psittacanthus robustus*

Acronym	Descriptions
Cadam	Incidence of the host tree *Campomanesia adamantium*
Dhirs	Incidence of the host tree *Diplusodon hirsutus*
Qcord	Incidence of the host tree *Qualea cordata*
Qdich	Incidence of the host tree *Qualea dichotoma*
Qgran	Incidence of the host tree *Qualea grandiflora*
Qmult	Incidence of the host tree *Qualea multiflora*
Qparv	Incidence of the host tree *Qualea parviflora*
Malbi	Incidence of the host tree *Miconia albicans*
Mferr	Incidence of the host tree *Miconia ferruginata*
Sconv	Incidence of the host tree *Salvertia convallariodora*
Tlani	Incidence of the host tree *Trembleya laniflora*
Vcinn	Incidence of the host tree *Vochysia cinnamomea*
Velli	Incidence of the host tree *Vochysia elliptica*
Vrufa	Incidence of the host tree *Vochysia rufa*
Vthyr	Incidence of the host tree *Vochysia thyrsoidea*
Vtuca	Incidence of the host tree *Vochysia tucanorum*
Ecris	Incidence of the seed-dispersing *Elaenia cristata*
Srufi	Incidence of the seed-dispersing *Schistochlamys ruficapillus*
Tviri	Incidence of the seed-dispersing *Tersina viridis viridis*
Tsaya	Incidence of the seed-dispersing *Thraupis sayaca sayaca*
bio2	Value of the mean diurnal range
bio3	Value of the isothermality
bio10	Value of the mean temperature of the warmest quarter
bio13	Value of the precipitation of the wettest month
bio15	Value of the precipitation seasonality
bio18	Value of the precipitation of the warmest quarter
bio19	Value of the precipitation of the coldest quarter

Binary classification models were constructed to predict mistletoe occurrence, with data split into training (80%) and testing (20%) subsets. Predictive models were implemented using ensemble algorithms, specifically Random Forest (Bagging), CatBoost, and XGBoost (Boosting) (Humphries et al. 2018). Model performance was evaluated on the test set using accuracy, precision, recall, and F1-score. Model optimization was performed through grid search combined with cross-validation, testing relevant hyperparameters for each algorithm. After validation, the Random Forest was selected as the best-performing algorithm and used to generate the final predictions. To interpret the model predictions, Shapley values (SHAP) were computed, providing global explanations of each variable’s contribution (Lundberg et al. 2020). Unlike Schoener’s D, which quantifies the degree of niche overlap between species (Warren et al. 2008), SHAP values quantify the contribution of each predictor variable to the model output, thereby indicating how each variable influences the predicted occurrence of *P. robustus* (Lundberg et al. 2020; Zbinden et al. 2026). The variables were ranked according to their mean absolute SHAP values, with higher values indicating a greater average contribution to model predictions. These analyses were conducted in Python using the libraries ‘pandas’ (https://pandas.pydata.org/), ‘numpy’ (Harris et al. 2020), ‘scikit-learn’ (Pedregosa et al. 2011), and ‘shap’ (Lundberg et al. 2020), and were executed in Jupyter Notebook.

## Results

### Species overlap in the environmental space

**Fig. 3:**
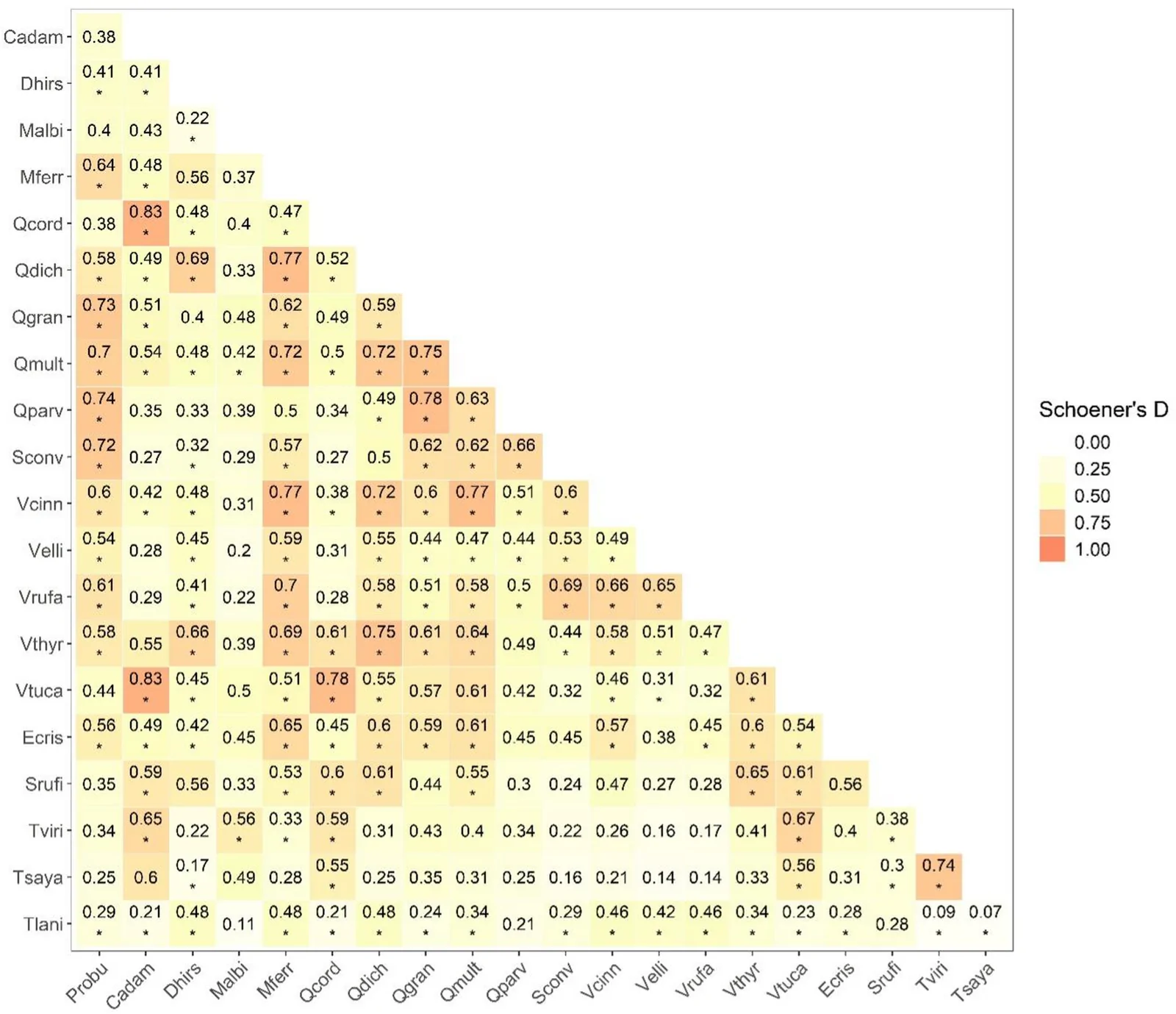
Observed values of Schoener’s D index for climatic niche overlap between each pair of species. The significance of the niche similarity test is shown below the D-index values (* = *p* < 0.05). Abbreviations: Probu= *Psittacanthus robustus*; Cadam= *Campomanesia adamantium*; Dhirs*= Diplusodon hirsutus*; Qcord= *Qualea cordata*; Qdich= *Qualea dichotoma*; Qgran= *Qualea grandiflora*; Qmult= *Qualea multiflora*, Qparv= *Qualea parviflora*; Malbi= *Miconia albicans*; Mferr= *Miconia ferruginata*; Sconv= *Salvertia convallariodora*; Tlani= *Trembleya laniflora*; Vcinn= *Vochysia cinnamomea*; Velli= *Vochysia elliptica*; Vrufa= *Vochysia rufa*; Vthyr= *Vochysia thyrsoidea*; Vtuca= *Vochysia tucanorum*; Ecris= *Elaenia cristata*; Srufi= *Schistochlamys ruficapillus*; Tviri= *Tersina viridis viridis*; Tsaya= *Thraupis sayaca sayaca*

The values of Schoener’s D index ranged from 0.07 to 0.83 (Fig. 3). *P. robustus* showed the highest values of niche overlap with host species (0.29–0.74) compared to seed-dispersing birds (0.25–0.56) (Fig. 3). The climatic niche of *P. robustus* was more similar than expected by chance to those of most host species and the bird *Elaenia cristata* (Fig. 3). However, the niche equivalence among *P. robustus*, hosts, and seed-disperser birds did not differ from a random pattern (Online Resource 1, Fig. S2).

### Occurrence predictors of the mistletoe

**Fig. 4:**
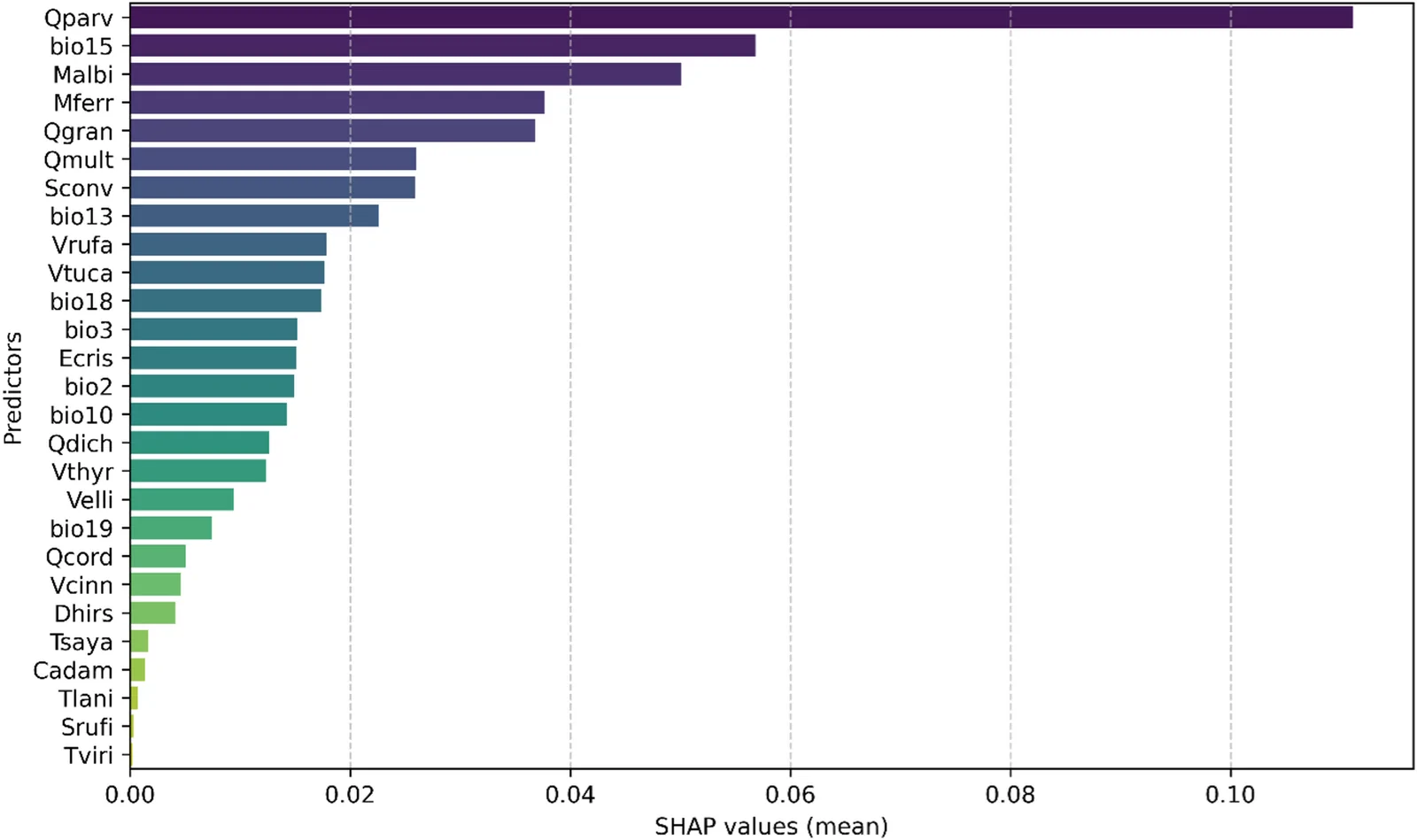
Global explanation (SHAP values) of the predictors of the model classification Random Forest, for the occurrence of the mistletoe *Psittacanthus robustus*. Refer to Table 1 for the abbreviations of the predictors

**Fig. 5:**
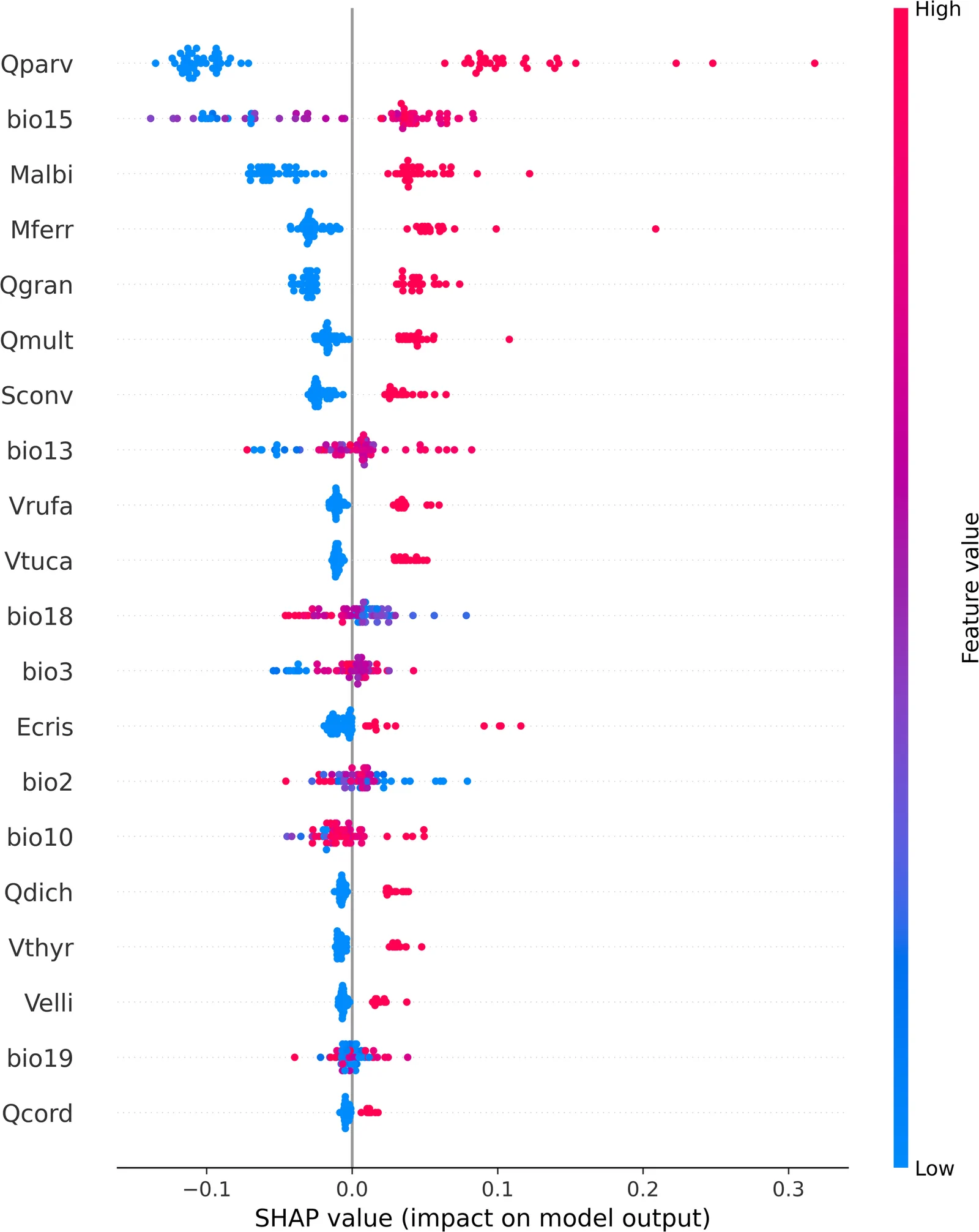
SHAP summary plot showing the relative importance and effect of predictors (local explanations) on the occurrence of *Psittacanthus robustus.* Each point represents the SHAP value for a given feature in one observation, with color indicating the feature value (red: high; blue: low). Positive SHAP values increase the probability of occurrence, whereas negative values decrease it. Refer to Table 1 for the abbreviations of the predictors

We found that Random Forest was the most effective algorithm for predicting the occurrence of *Psittacanthus robustus* (Online Resource 1, Table S4). Model performance differed between classes, with higher predictive accuracy for absences (precision = 0.91, recall = 0.91, F1-score = 0.91) than for presences (precision = 0.91, recall = 0.90, F1-score = 0.90). The SHAP values analysis revealed that the host tree *Qualea parviflora* was the strongest predictor of *P. robustus* occurrence (Fig. 4). Precipitation seasonality, along with the host trees *Miconia albicans* and *Miconia ferruginata*, completed the set of the four most important variables (Fig. 4). Positive SHAP values of the host species were associated with high feature values, indicating that the presence of these trees increases the probability of occurrence of *P. robustus*. Also, negative SHAP values of the host species were associated with low feature values, indicating that the absence or low occurrence of these trees reduces the probability of occurrence of *P. robustus* (Fig. 5). We also identified that these variables were strongly determinant and had almost linear effects on the prediction of *P. robustus* (Fig. 5). Generally, high precipitation seasonality increased the probability of occurrence of *P. robustus* (Fig. 5). Other climatic variables showed a similar or less consistent pattern in predicting the absence and presence of *P. robustus* (Fig. 5). Despite its low importance value, the seed-dispersing bird *Elaenia cristata* exhibited a pattern similar to that of the host trees, with its occurrence being positively associated with the predicted occurrence of *P*. *robustus* (Figs. 4 and 5).

## Discussion

We found a higher overlap of the climatic niche between *P. robustus* and the host trees than among the seed-dispersing birds. Although the climatic niche of *P. robustus* was like that of most species, we did not detect niche equivalence. As expected, host trees exerted a primary effect on the spatial occurrence of *P. robustus*, particularly *Q. parviflora*. The climatic predictors exhibited a secondary effect on the incidence of *P. robustus*, mainly precipitation seasonality. With a comparatively lower contribution to the model, the spatial incidence of the seed-dispersing bird *Elaenia cristata* was also able to predict the presence and absence of *P. robustus*.

We found higher niche similarity between *P. robustus* and the host tree than with seed-dispersing birds. As parasites, mistletoes should be integrated hydraulically and nutritionally with a compatible host to persist (Wang et al. 2022, 2023). While mistletoe species are spread by overlapping groups of frugivorous animals, the birds responsible for seed dispersal in this study interact with a variety of plants during their feeding activities (Guerra and Pizo 2014). For instance, although it has been identified as one of the main dispersers of *P*. *robustus* (Monteiro et al. 1992), *T*. *viridis viridis* feeds on other mistletoe species, such as *Struthanthus concinnus* (Mart.) Mart. and *Struthanthus marginatus* (Desr.) Blume, as well as members of other families, including Magnoliaceae and Araliaceae (Guerra and Pizo 2014; Hilty 2020). Furthermore, niche dissimilarity is also caused by the great distribution range of dispersing animals, resulting in a broader climatic niche, as in the case of the birds evaluated in this study (Guisan and Thuiller 2005; Rubim 2009).

Although niche convergences were detected, we found that the climatic niches of the species were not identical. These combined results suggest that infection is restricted to a portion of the host niche that overlaps the *P. robustus* niche, which occurs under particular climatic conditions of the host trees (Lira-Noriega and Peterson 2014). This also suggests that the occurrence of mistletoe is not exclusively determined by interactions with hosts and seed dispersers but also by climatic constraints (Ramirez-Barahona et al. 2017). Thus, the realized niche of *P. robustus* is constrained by the intersection of climatic suitability, host occurrence, and seed disperser occurrence, resulting in a narrower environmental space than that occupied by its hosts or dispersers individually (Teodoro et al. 2010; Ramírez-Barahona et al. 2017; Ojeda et al. 2023). This pattern is consistent with the parasitological theory, which predicts that parasite distributions emerge from the interaction between abiotic constraints, environmental filters, and host compatibility and availability, rather than from climatic conditions alone (Kuhn et al. 2016; Klimov and He 2024). Accordingly, parasite distributions rarely coincide with their potential range and are restricted to a subset of the environmental space occupied by their hosts (Huang et al. 2018; Matthies 2021).

Our results revealed that host tree identity explains mistletoe distribution better than abiotic climate or dispersal vectors, such as birds. The establishment of mistletoe on the host depends on physiological correspondence, limiting infection even under favorable climatic conditions and adequate dispersion (Wang et al. 2023; Rödl and Ward 2002). While birds influence where the seeds are deposited, they alone do not ensure germination, since physiological incompatibility can nullify the seed rain (Okubamichael et al. 2016). In a similar way, climate influences reproductive performance and timing but does not remove the fundamental constraint of host dependence (Watson 2001; Aizen 2003). Thus, host identity acts as an establishment filter (Rödl and Ward 2002), manifesting in the co-occurrence patterns of *P. robustus* (Araujo and Rozenfeld 2014).

*Psittacanthus robustus* tends to occur in regions with pronounced precipitation seasonality. Climates marked by distinct seasons establish moisture windows that allow mistletoe infection. These periods are characterized by non-desiccating conditions and benign temperatures, essential for seed germination and successful haustorial penetration (Rödl and Ward 2002; Aizen 2003). This may be partly explained by the synchronization between fruiting phenology and disperser activity (Aizen 2003; Mellado and Zamora 2016). Furthermore, seasonal stress may benefit mistletoe establishment by increasing host susceptibility to infection (Medel et al. 2004; Mellado and Zamora 2016) and suppressing potential competitors, such as fungi and epiphytes (Wang et al. 2022, 2023). These explanations are consistent with an increasing occurrence under moderate to high seasonality. However, very high seasonality has the potential to limit host water supply and reduce mistletoe survival (Watson 2001; Wang et al. 2022, 2023). Thus, the relationship between climatic predictors and mistletoe occurrence is highly unlikely to be linear.

Despite the generally weak influence of seed-dispersing birds in our models, the presence of *E*. *cristata* enhanced the probability of *P*. *robustus* occurrence. Its influence was even stronger than that of the host tree species from the genera *Qualea* spp. and *Vochysia* spp. This pattern contrasts with earlier reports identifying *T. viridis viridis* as the main seed disperser of *P. robustus* (Monteiro et al. 1992). *E. cristata*, a member of the Tyrannidae, has an overall diet composed of approximately 60% arthropods and 40% fruits (Şekercioğlu et al. 2025). However, vertebrate diets vary according to local resource availability, and *E. cristata* may exhibit a highly frugivorous diet. For instance, Guerra and Pizo (2014) reported that fruits comprised 77% of its diet, with *P. robustus* being the second most frequently consumed plant species among the 12 identified. Generalist frugivores can move among different host trees and habitat patches while exploiting multiple food resources, potentially increasing opportunities for the establishment of new infection sites (Watson 2013). Although this hypothesis remains to be tested for *P. robustus*, it provides an ecological explanation for the importance of *E. cristata* in our macroecological models.

Evidence indicates a strong ecological association between *E. cristata* and *P. robustus*. Guerra (2010) found that 94% of the feeding bouts recorded for *E. cristata* involved fruits of *P. robustus*. Seeds were subsequently dispersed away from the parent plant and deposited on other host trees, confirming the effectiveness of *E. cristata* as a seed disperser. This high dispersal efficiency results from the ingestion of whole mistletoe fruits, followed by obligatory seed regurgitation. The adhesive seeds retain their viscosity and often adhere to the bird’s bill, prompting bill-wiping behavior that promotes effective deposition on suitable host branches (Guerra 2010). In addition, anatomical differences between *E. cristata* and *T. viridis viridis* likely underpin their contrasting dietary patterns. *E. cristata* preferentially consumes small fruits (Guerra and Pizo 2014), whereas the broader beak of *T. viridis viridis* enables it to handle and specialize on larger fruits, such as *Nectandra megapotamica* (Lauraceae), consuming mistletoes only opportunistically (Rubim 2009; Hilty 2020). These morphological and dietary distinctions may contribute to differences in their seed-dispersal roles. Nevertheless, our analyses do not quantify dietary breadth or seed-dispersal effectiveness and therefore do not establish that *E. cristata* is a more effective disperser than *T. viridis viridis*. The relative contribution of these species may vary geographically as a consequence of spatial variation in disperser abundance, which influences the frequency of interactions with *P. robustus* (Guerra 2010; Fowler et al. 2023).

Overall, our findings highlight the importance of biotic interactions in shaping the macroecological distribution of *P. robustus*. Host trees exerted the strongest influence on its occurrence, while climatic conditions and seed-dispersing birds provided secondary contributions. The observed niche similarity, coupled with the lack of niche equivalence, indicates that the realized distribution of *P. robustus* emerges from the combined effects of climatic suitability, host availability, and seed dispersers. Our dataset reflects the current state of knowledge on seed dispersers rather than the complete disperser assemblage across the species’ distribution. Thus, regionally important dispersers may remain undocumented, and geographic turnover in disperser species is likely across the range of *P. robustus*. Finally, our study reinforces the importance of incorporating biotic interactions into species distribution models. Such an approach should provide a more realistic understanding of parasite distributions than models based solely on abiotic predictors.

## Supplementary Information

Below is the link to the electronic supplementary material.

Supplementary Material 1

## Data Availability

The incidence matrix of the species investigated used on machine-learning classification models can be accessed in Online Resource 1, Table S3. Further data will be provided by the corresponding author upon request.

## Code availability

The data, scripts (.R) and Notebooks (.ipynb) used in this study are available at: https://github.com/EduvOliveira/Machine-learning_mistletoe/tree/main.
