# Supplementary material for: Disentangling biotic and abiotic drivers of a neotropical mistletoe
Source: Oecologia. 2026 Sep 15;208(10):137. doi: 10.1007/s00442-026-05975-6 (PMC13577974; doi:10.1007/s00442-026-05975-6)
Supplement: Supplementary file 1 [file 442_2026_5975_MOESM1_ESM.pdf]

Oecologia

Online Resource 1

Disentangling biotic and abiotic drivers of a neotropical mistletoe

Eduardo V. S. Oliveira[1*], Thieres Santos Almeida[2], Helon Simões Oliveira[2], Sidney F. Gouveia[1]

[1] Department of Ecology, Federal University of Sergipe, Sergipe, Brazil. ORCID id 0000-0003-4858-3930 (E.V.S.O.); ORCID id 0000-0003-4941-7440 (S.F.G.)

[2] Graduate program in Ecology and Conservation, Federal University of Sergipe, São Cristóvão, Sergipe, Brazil. ORCID id 0000-0001-7371-8070 (T.S.A.); ORCID id 0000-0002-5548-2698 (H.S.O.)

* Corresponding author: eduardovso@yahoo.com.br

Table S1 Host trees and seed-dispersing bird species of *Psittacanthus robustus* compiled from a literature review.

	FAMILY/Specie
Host trees	LYTHRACEAE
	Diplusodon hirsutus (Cham. & Schltdl.) A.DC. [1]
	MELASTOMATACEAE
	Miconia albicans (Sw.) Steud. [1, 2]
	Miconia ferruginata DC. [1]
	Trembleya laniflora (D.Don) Cogn. [1]
	MYRTACEAE
	Campomanesia adamantium (Cambess.) O.Berg [1]
	POLYGONACEAE
	Coccoloba cereifera Schwacke [1]
	VOCHYSIACEAE
	Salvertia convallariodora A.St.-Hil. [2]
	Qualea cordata Spreng. [1, 2]
	Qualea dichotoma (Mart.) Warm. [1, 2]
	Qualea grandiflora Mart. [2]
	Qualea multiflora Mart. [2]
	Qualea parviflora Mart. [2]
	Vochysia cinnamomea Pohl [2]
	Vochysia elliptica Mart. [1, 2]
	Vochysia rufa Mart. [2]
	Vochysia tucanorum Mart [2]
	Vochysia thyrsoidea Pohl. [1, 2, 4, 5, 6, 7, 8]

	FAMILY/Specie
Seed-dispersing birds	THRAUPIDAE
	Tersina viridis viridis (Illiger, 1811) [1, 2, 4, 5, 8, 9]
	Thraupis sayaca sayaca (Linnaeus, 1766) [2, 9]
	Schistochlamys ruficapillus (Vieillot, 1817) [2, 9]
	TYRANNIDAE
	Elaenia cristata Pelzeln, 1868 [3, 4, 9]

Source:

[1] Guerra TJ, Pizo MA, Silva WR (2018) Host specificity and aggregation for a widespread mistletoe in Campo Rupestre vegetation. Flora 238:148–154.

[2] Monteiro RF, Martins RP, Yamamoto K (1992) Host Specificity and Seed Dispersal of *Psittacanthus robustus* (Loranthaceae) in South-East Brazil. J Trop Ecol 8:307–314.

[3] Guerra TJ, Pizo MA (2014) Asymmetrical Dependence Between a Neotropical Mistletoe and its Avian Seed Disperser. Biotropica 46:285–293.

[4] Teodoro GS, Berg E, Santos MCN & Coelho FF (2010) How does a *Psittacanthus robustus* Mart. population structure relate to a *Vochysia thyrsoidea* Pohl. host population? Flora 205: 797–801.

[5] Arruda R, Fadini RF, Carvalho LN, Del-Claro K, Mourão FA, Jacobi CM et al. (2012). Ecology of neotropical mistletoes: an important canopy-dwelling component of Brazilian ecosystems. Acta Bot. Bras. 26:264-274.

[6] Teodoro GS, van den Berg E, Arruda R (2013) Metapopulation dynamics of the mistletoe and its host in savanna areas with different fire occurrence. PLoS One 8:e65836.

[7] Silva MC, Guimarães AF, Teodoro GS, Bastos SS, de Castro EM, van den Berg E (2021) The enemy within: the effects of mistletoe parasitism on infected and uninfected host branches. Plant Ecol. 222:639-645.

[8] Arruda R, Fadini RF, Mourão FA, Jacobi CM, Teodoro GS (2009) Natural history and ecology of Neotropical mistletoes. Tropical Biology and Conservation Management: Natural history of tropical plants. Oxford, Eolss Publishers, 133-154.

[9] Guerra TJA (2010) História natural da erva-de-passarinho *Psittacanthus robustus* (Loranthaceae) em uma área de campo rupestre do sudeste brasileiro: interações com hospedeiras, dispersores, polinizadores e insetos herbívoros. Doctoral Thesis, State University of Campinas, Brazil.

Table S2 List of codes and descriptions of the 19 bioclimatic variables obtained from the WorldClim database used in the performed analyses (O'Donnel and Ignizio 2012; Fick and Hijmans 2017).

Code	Description
BIO01	Annual Mean Temperature
BIO02	Mean Diurnal Range
BIO03	Isothermality (BIO02/BIO07) (×100)
BIO04	Temperature Seasonality
BIO05	Max Temperature of Warmest Month
BIO06	Min Temperature of Coldest Month
BIO07	Temperature Annual Range (BIO05 – BIO06)
BIO08	Mean Temperature of Wettest Quarter
BIO09	Mean Temperature of Driest Quarter
BIO10	Mean Temperature of Warmest Quarter
BIO11	Mean Temperature of Coldest Quarter
BIO12	Annual Precipitation
BIO13	Precipitation of Wettest Month
BIO14	Precipitation of Driest Month
BIO15	Precipitation Seasonality
BIO16	Precipitation of the Wettest Quarter
BIO17	Precipitation of the Driest Quarter
BIO18	Precipitation of Warmest Quarter
BIO19	Precipitation of Coldest Quarter

References:
O'Donnel, M. S., & Ignizio, D. A. (2012). Bioclimatic predictors for supporting ecological applications in the conterminous United States (No. 691). US Geological Survey.
Fick, S. E., & Hijmans, R. J. (2017). WorldClim 2: new 1-km spatial resolution climate surfaces for global land areas. International journal of climatology, 37(12), 4302-4315.

Table S3 Incidence matrix for *P. robustus*, hosts, and seed-disperser birds obtained from occurrence records of species (buffers collapsed) overlaid by a grid cells (110 × 110 km; ~1°) of South America. Abbreviations: Probu= *Psittacanthus robustus*; Cadam= *Campomanesia adamantium*; *Diplusodon hirsutus*= Dhirs; *Qualea cordata*= Qcord; Qdich= *Qualea dichotoma*; Qgran= *Qualea grandiflora*; Qmult= *Qualea multiflora*, Qparv= *Qualea parviflora*; *Miconia albicans*= Malbi; *Miconia ferruginata*= Mferr; Sconv= *Salvertia convallariodora*; *Trembleya laniflora*= Tlani; Vcinn= *Vochysia cinnamomea*; Velli= *Vochysia elliptica*; Vrufa= *Vochysia rufa*; Vthyr= *Vochysia thyrsoidea*; Vtuca= *Vochysia tucanorum*; Ecris= *Elaenia cristata*; Srufi= *Schistochlamys ruficapillus*; Tviri= *Tersina viridis viridis*; Tsaya= *Thraupis sayaca sayaca*.

Long(x)	Lat(y)	Probu	Cadam	Dhirs	Ecris	Malbi	Mferr	Qcord	Qdich	Qgran	Qmult	Qparv	Sconv	Srufi	Tviri	Tsaya	Tlani	Vcinn	Velli	Vrufa	Vthyr	Vtuca
-59.90	2.27	0	0	0	1	1	0	0	0	0	0	0	0	0	0	0	0	0	0	0	0	0
-35.50	-7.50	0	0	0	1	1	0	0	0	0	0	0	0	0	0	0	0	0	0	0	0	0
-55.50	-21.50	0	0	0	0	0	0	0	0	1	0	0	0	0	0	1	0	0	0	0	0	0
-70.50	-17.50	0	0	0	0	0	0	0	0	0	0	0	0	0	0	0	0	0	0	0	0	0
-75.99	-2.01	0	0	0	0	0	0	0	0	0	0	0	0	0	0	0	0	0	0	0	0	0
-61.50	3.50	0	0	0	1	0	0	0	0	0	0	0	0	0	0	0	0	0	0	0	0	0
-71.46	10.57	0	0	0	0	1	0	0	0	0	0	0	0	0	0	0	0	0	0	0	0	0
-45.56	-1.65	0	0	0	0	0	0	0	0	0	0	0	0	0	0	0	0	0	0	0	0	0
-56.50	-17.50	0	0	0	0	0	0	0	0	0	0	1	0	0	0	0	0	0	0	1	0	0
-73.51	-5.49	0	0	0	0	0	0	0	0	0	0	0	0	0	0	0	0	0	0	0	0	0
-78.20	-3.69	0	0	0	0	1	0	0	0	0	0	0	0	0	0	0	0	0	0	0	0	0
-74.50	1.50	0	0	0	0	1	0	0	0	0	0	0	0	0	0	0	0	0	0	0	0	0
-66.51	-47.52	0	0	0	0	0	0	0	0	0	0	0	0	0	0	0	0	0	0	0	0	0
-60.50	-0.50	0	0	0	0	0	0	0	0	0	0	0	0	0	0	0	0	0	0	0	0	0
-70.50	-41.50	0	0	0	0	0	0	0	0	0	0	0	0	0	0	0	0	0	0	0	0	0
-71.50	2.50	0	0	0	0	0	0	0	0	0	0	0	0	0	0	0	0	0	0	0	0	0
-57.50	-5.50	0	0	0	0	0	0	0	0	0	0	0	0	0	0	0	0	0	0	0	0	0
-48.50	-9.50	0	0	0	1	1	1	0	0	0	1	1	1	0	0	0	0	1	1	0	0	0
-51.50	-27.50	0	0	0	0	0	0	0	0	0	0	0	0	0	0	0	0	0	0	0	0	0

Long(x)	Lat(y)	Probu	Cadam	Dhirs	Ecris	Malbi	Mferr	Qcord	Qdich	Qgran	Qmult	Qparv	Sconv	Srufi	Tvir i	Tsaya	Tlani	Vcinn	Vell i	Vrufa	Vthyr	Vtuca
-62.50	0.50	0	0	0	0	0	0	0	0	0	0	0	0	0	0	0	0	0	0	0	0	0
-51.50	-2.51	0	0	0	0	0	0	0	0	0	0	0	0	0	0	0	0	0	0	0	0	0
-66.50	-16.50	0	0	0	0	0	0	0	0	0	0	0	0	0	0	0	0	0	0	0	0	0
-61.50	-25.50	0	0	0	0	0	0	0	0	0	0	0	0	0	0	0	0	0	0	0	0	0
-61.50	-26.50	0	0	0	0	0	0	0	0	0	0	0	0	0	0	0	0	0	0	0	0	0
-75.50	-8.50	0	0	0	0	0	0	0	0	0	0	0	0	0	0	0	0	0	0	0	0	0
-70.50	-11.51	0	0	0	0	1	0	0	0	1	1	0	0	0	0	0	0	0	0	0	0	0
-66.50	-27.50	0	0	0	0	0	0	0	0	0	0	0	0	0	0	0	0	0	0	0	0	0
-68.49	6.09	0	0	0	0	1	0	0	0	0	0	0	0	0	0	0	0	0	0	0	0	0
-56.50	4.50	0	0	0	1	0	0	0	0	0	0	0	0	0	0	0	0	0	0	0	0	0
-72.50	-7.50	0	0	0	0	0	0	0	0	0	0	0	0	0	0	0	0	0	0	0	0	0
-65.50	-38.50	0	0	0	0	0	0	0	0	0	0	0	0	0	0	0	0	0	0	0	0	0
-54.71	4.48	0	0	0	0	0	0	0	0	0	0	0	0	0	0	0	0	0	0	0	0	0
-75.50	6.50	0	0	0	0	1	0	0	0	0	0	0	0	0	0	0	0	0	0	0	0	0
-59.29	1.74	0	0	0	0	0	0	0	0	0	0	0	0	0	0	0	0	0	0	0	0	0
-57.95	-20.03	0	0	0	0	0	0	0	0	1	0	0	0	0	0	0	0	0	0	0	0	0
-76.50	-7.50	0	0	0	0	0	0	0	0	1	0	0	0	0	0	0	0	0	0	0	0	0
-58.50	5.50	0	0	0	0	0	0	0	0	0	0	0	0	0	0	0	0	0	0	0	0	0
-58.50	-29.50	0	0	0	0	0	0	0	0	0	0	0	0	0	0	1	0	0	0	0	0	0
-64.39	1.25	0	0	0	0	0	0	0	0	0	0	0	0	0	0	0	0	0	0	0	0	0
-56.50	-12.50	0	0	0	0	1	1	0	1	1	1	1	1	0	0	0	0	1	0	0	0	1
-72.47	-5.52	0	0	0	0	0	0	0	0	0	0	0	0	0	0	0	0	0	0	0	0	0
-76.41	-2.68	0	0	0	0	0	0	0	0	0	0	0	0	0	0	0	0	0	0	0	0	0
-71.85	-51.95	0	0	0	0	0	0	0	0	0	0	0	0	0	0	0	0	0	0	0	0	0
-64.50	-29.50	0	0	0	0	0	0	0	0	0	0	0	0	0	0	0	0	0	0	0	0	0
-57.61	5.52	0	0	0	0	1	0	0	0	0	0	0	0	0	0	0	0	0	0	0	0	0
-56.50	-10.50	0	0	0	0	1	0	0	0	1	0	1	1	0	0	0	0	0	0	0	0	0
-57.14	-29.88	0	0	0	0	0	0	0	0	0	0	0	0	0	0	0	0	0	0	0	0	0

Long(x)	Lat(y)	Probu	Cadam	Dhirs	Ecris	Malbi	Mferr	Qcord	Qdich	Qgran	Qmult	Qparv	Sconv	Srufi	Tviri	Tsaya	Tlani	Vcinn	Velli	Vrufa	Vthyr	Vtuca
-59.50	6.50	0	0	0	0	0	0	0	0	0	0	0	0	0	0	0	0	0	0	0	0	0
-57.82	-30.37	0	0	0	0	0	0	0	0	0	0	0	0	0	0	1	0	0	0	0	0	0
-61.50	0.50	0	0	0	0	0	0	0	0	0	0	0	0	0	0	0	0	0	0	0	0	0
-61.56	-19.35	0	0	0	0	0	0	0	0	0	0	0	0	0	0	0	0	0	0	0	0	0
-69.96	-4.09	0	0	0	0	0	0	0	0	0	0	0	0	0	0	0	0	0	0	0	0	0
-67.50	-17.50	0	0	0	0	0	0	0	0	0	0	0	0	0	0	0	0	0	0	0	0	0
-55.27	-31.12	0	0	0	0	0	0	0	0	0	0	0	0	0	0	0	0	0	0	0	0	0
-39.50	-15.50	0	0	0	0	1	1	1	0	0	0	0	0	0	0	1	0	1	0	1	0	0
-55.50	-13.50	0	0	0	0	1	0	0	1	1	1	1	0	0	0	0	0	1	0	0	0	1
-55.50	0.50	0	0	0	0	0	0	0	0	0	0	0	0	0	0	0	0	0	0	0	0	0
-52.50	-6.50	0	0	0	0	0	0	0	0	0	0	1	0	0	0	0	0	0	0	0	0	0
-69.92	-18.10	0	0	0	0	0	0	0	0	0	0	0	0	0	0	0	0	0	0	0	0	0
-60.50	-20.50	0	0	0	0	0	0	0	0	1	1	1	0	0	0	0	0	0	0	0	0	1
-73.05	-2.21	0	0	0	0	0	0	0	0	0	0	0	0	0	0	0	0	0	0	0	0	0
-63.50	-14.50	0	0	0	0	0	0	0	0	0	0	0	0	0	0	0	0	0	0	0	0	0
-55.50	-32.50	0	0	0	0	0	0	0	0	0	0	0	0	0	0	0	0	0	0	0	0	0
-67.59	-10.27	0	0	0	0	0	0	0	0	1	0	0	0	0	0	0	0	0	0	0	0	0
-60.50	-32.50	0	0	0	0	0	0	0	0	0	0	0	0	0	0	0	0	0	0	0	0	0
-69.50	-25.50	0	0	0	0	0	0	0	0	0	0	0	0	0	0	0	0	0	0	0	0	0
-69.59	0.82	0	0	0	0	0	0	0	0	0	0	0	0	0	0	0	0	0	0	0	0	0
-66.50	-15.50	0	0	0	0	1	0	0	0	0	0	0	0	0	0	0	0	0	0	0	0	0
-67.06	-22.80	0	0	0	0	0	0	0	0	0	0	0	0	0	0	0	0	0	0	0	0	0
-68.59	-21.53	0	0	0	0	0	0	0	0	0	0	0	0	0	0	0	0	0	0	0	0	0
-67.50	-28.50	0	0	0	0	0	0	0	0	0	0	0	0	0	0	0	0	0	0	0	0	0
-77.20	3.39	0	0	0	0	0	0	0	0	0	0	0	0	0	0	0	0	0	0	0	0	0
-69.46	-55.18	0	0	0	0	0	0	0	0	0	0	0	0	0	0	0	0	0	0	0	0	0
-57.50	-3.50	0	0	0	0	0	0	0	0	0	0	0	0	0	0	0	0	0	0	0	0	0
-72.05	-42.61	0	0	0	0	0	0	0	0	0	0	0	0	0	0	0	0	0	0	0	0	0

Long(x)	Lat(y)	Probu	Cadam	Dhirs	Ecris	Malbi	Mferr	Qcord	Qdich	Qgran	Qmult	Qparv	Sconv	Srufi	Tviri	Tsaya	Tlani	Vcinn	Velli	Vrufa	Vthyr	Vtuca
-63.50	-33.50	0	0	0	0	0	0	0	0	0	0	0	0	0	0	0	0	0	0	0	0	0
-52.14	3.20	0	0	0	0	1	0	0	0	0	0	0	0	0	0	0	0	0	0	0	0	0
-71.57	-37.50	0	0	0	0	0	0	0	0	0	0	0	0	0	0	0	0	0	0	0	0	0
-62.50	-6.50	0	0	0	0	0	0	0	0	0	0	0	0	0	0	0	0	0	0	0	0	0
-79.50	-0.50	0	0	0	0	0	0	0	0	0	0	0	0	0	0	0	0	0	0	0	0	0
-44.50	-9.50	0	0	0	0	0	0	0	0	1	1	1	0	0	0	0	0	0	0	0	0	0
-69.32	-2.52	0	0	0	0	0	0	0	0	0	0	0	0	0	0	0	0	0	0	0	0	0
-49.50	-12.50	0	0	0	0	0	0	0	0	1	1	1	1	0	0	0	0	0	0	1	0	0
-53.50	-14.50	0	0	0	0	0	0	0	0	0	0	0	0	0	0	0	0	0	0	0	0	0
-68.32	-54.42	0	0	0	0	0	0	0	0	0	0	0	0	0	0	0	0	0	0	0	0	0
-63.50	1.50	0	0	0	0	0	0	0	0	0	0	0	0	0	0	0	0	0	0	0	0	0
-38.50	-8.50	0	0	0	0	1	0	0	0	0	0	0	0	0	0	0	0	0	0	0	0	0
-53.85	-26.50	0	0	0	0	0	0	0	0	0	0	0	0	0	1	1	0	0	0	0	0	0
-63.47	-41.08	0	0	0	0	0	0	0	0	0	0	0	0	0	0	0	0	0	0	0	0	0
-75.50	-4.50	0	0	0	0	0	0	0	0	0	0	0	0	0	0	0	0	0	0	0	0	0
-39.50	-12.50	0	0	0	0	1	0	0	0	0	0	0	0	0	0	0	0	0	0	0	1	0
-44.50	-10.50	0	0	0	0	0	1	0	1	1	0	1	1	0	0	1	0	0	0	0	0	0
-72.49	-52.49	0	0	0	0	0	0	0	0	0	0	0	0	0	0	0	0	0	0	0	0	0
-65.50	-25.50	0	0	0	0	0	0	0	0	0	0	0	0	0	0	0	0	0	0	0	0	0
-77.50	-3.50	0	0	0	0	0	0	0	0	0	0	0	0	0	0	0	0	0	0	0	0	0
-72.09	-10.00	0	0	0	0	0	0	0	0	0	0	0	0	0	0	0	0	0	0	0	0	0
-65.50	-34.50	0	0	0	0	0	0	0	0	0	0	0	0	0	0	0	0	0	0	0	0	0
-57.50	-14.50	0	0	0	0	1	1	0	0	1	1	1	0	0	0	0	0	1	0	1	0	0
-67.50	-2.50	0	0	0	0	0	0	0	0	0	0	0	0	0	0	0	0	0	0	0	0	0
-61.50	7.50	0	0	0	0	1	0	0	0	0	0	0	0	0	0	0	0	0	0	0	0	0
-71.53	-38.47	0	0	0	0	0	0	0	0	0	0	0	0	0	0	0	0	0	0	0	0	0
-54.50	-11.50	0	0	0	0	0	0	0	0	0	0	0	0	0	0	0	0	0	0	0	0	0
-76.50	1.50	0	0	0	0	0	0	0	0	0	0	0	0	0	0	0	0	0	0	0	0	0

Long(x)	Lat(y)	Probu	Cadam	Dhirs	Ecris	Malbi	Mferr	Qcord	Qdich	Qgran	Qmult	Qparv	Sconv	Srufi	Tviri	Tsaya	Tlani	Vcinn	Velli	Vrufa	Vthyr	Vtuca
-62.50	1.50	0	0	0	0	0	0	0	0	0	0	0	0	0	0	0	0	0	0	0	0	0
-63.50	-4.50	0	0	0	0	0	0	0	0	0	0	0	0	0	0	0	0	0	0	0	0	0
-40.50	-16.50	0	0	0	0	1	0	0	0	0	0	0	0	0	0	0	0	0	0	0	0	0
-58.50	-15.50	0	0	0	0	0	0	0	0	0	0	1	0	0	0	0	0	0	0	1	0	0
-35.72	-9.33	0	0	0	1	1	1	0	0	0	0	0	0	0	0	1	0	0	0	0	0	0
-65.50	-32.50	0	0	0	0	0	0	0	0	0	0	0	0	0	0	0	0	0	0	0	0	0
-68.50	-49.49	0	0	0	0	0	0	0	0	0	0	0	0	0	0	0	0	0	0	0	0	0
-73.52	-2.51	0	0	0	0	0	0	0	0	0	0	0	0	0	0	0	0	0	0	0	0	0
-38.73	-3.80	0	0	0	1	0	0	0	0	0	0	0	0	0	0	1	0	0	0	0	0	0
-72.50	-0.50	0	0	0	0	0	0	0	0	0	0	0	0	0	0	0	0	0	0	0	0	0
-71.38	-44.48	0	0	0	0	0	0	0	0	0	0	0	0	0	0	0	0	0	0	0	0	0
-70.25	-31.48	0	0	0	0	0	0	0	0	0	0	0	0	0	0	0	0	0	0	0	0	0
-60.50	-6.50	0	0	0	0	0	0	0	0	0	0	0	0	0	0	0	0	0	0	0	0	0
-81.08	-6.07	0	0	0	0	0	0	0	0	0	0	0	0	0	0	0	0	0	0	0	0	0
-70.14	-30.74	0	0	0	0	0	0	0	0	0	0	0	0	0	0	0	0	0	0	0	0	0
-53.50	3.50	0	0	0	0	1	0	0	0	0	0	0	0	0	0	0	0	0	0	0	0	0
-63.50	-5.50	0	0	0	0	0	0	0	0	0	0	0	0	0	0	0	0	0	0	0	0	0
-65.50	-17.50	0	0	0	0	0	0	0	0	0	0	0	0	0	0	0	0	0	0	0	0	0
-56.76	1.95	0	0	0	0	1	0	0	0	0	0	0	1	0	0	0	0	0	0	0	0	0
-69.31	-10.98	0	0	0	0	0	0	0	0	0	0	0	0	0	0	0	0	0	0	0	0	0
-76.50	4.50	0	0	0	0	0	0	0	0	0	0	0	0	0	0	0	0	0	0	0	0	0
-68.50	-34.50	0	0	0	0	0	0	0	0	0	0	0	0	0	0	0	0	0	0	0	0	0
-57.53	2.48	0	0	0	0	0	0	0	0	0	0	0	0	0	0	0	0	0	0	0	0	0
-71.32	-30.55	0	0	0	0	0	0	0	0	0	0	0	0	0	0	0	0	0	0	0	0	0
-45.50	-19.50	0	1	0	1	1	0	1	0	1	0	0	1	0	0	0	1	0	0	0	1	0
-76.50	-6.50	0	0	0	0	1	0	0	0	0	0	0	0	0	0	0	0	0	0	0	0	0
-67.50	-4.50	0	0	0	0	0	0	0	0	0	0	0	0	0	0	0	0	0	0	0	0	0
-70.04	-33.13	0	0	0	0	0	0	0	0	0	0	0	0	0	0	0	0	0	0	0	0	0

Long(x)	Lat(y)	Probu	Cadam	Dhirs	Ecris	Malbi	Mferr	Qcord	Qdich	Qgran	Qmult	Qparv	Sconv	Srufi	Tviri	Tsaya	Tlani	Vcinn	Velli	Vrufa	Vthyr	Vtuca
-48.85	-28.27	0	0	0	0	0	0	0	0	0	0	0	0	0	0	0	0	0	0	0	0	0
-72.46	-36.52	0	0	0	0	0	0	0	0	0	0	0	0	0	0	0	0	0	0	0	0	0
-59.50	-5.50	0	0	0	0	0	0	0	0	0	0	0	0	0	1	0	0	0	0	0	0	0
-53.50	-4.50	0	0	0	0	0	0	0	0	0	0	0	0	0	0	0	0	0	0	0	0	0
-71.50	-7.50	0	0	0	0	0	0	0	0	0	0	0	0	0	0	0	0	0	0	0	0	0
-69.50	-13.50	0	0	0	0	1	0	0	0	1	1	0	0	0	0	0	0	0	0	0	0	0
-73.35	-1.36	0	0	0	0	0	0	0	0	0	0	0	0	0	0	0	0	0	0	0	0	0
-70.49	7.52	0	0	0	0	1	0	0	0	0	0	0	0	0	0	0	0	0	0	0	0	0
-72.50	-15.50	0	0	0	0	0	0	0	0	0	0	0	0	0	0	0	0	0	0	0	0	0
-68.24	-26.47	0	0	0	0	0	0	0	0	0	0	0	0	0	0	0	0	0	0	0	0	0
-68.33	-19.46	0	0	0	0	0	0	0	0	0	0	0	0	0	0	0	0	0	0	0	0	0
-64.50	-28.50	0	0	0	0	0	0	0	0	0	0	0	0	0	0	0	0	0	0	0	0	0
-57.43	-22.09	0	1	0	0	1	0	1	0	1	0	1	0	0	0	0	0	0	0	0	0	1
-49.50	-0.56	0	0	0	0	0	0	0	0	0	0	0	0	0	0	0	0	0	0	0	0	0
-62.50	6.50	0	0	0	0	1	0	0	0	0	0	0	0	0	0	0	0	0	0	0	0	0
-49.52	-1.45	0	1	0	0	0	0	0	0	0	0	0	0	0	0	0	0	0	0	0	0	0
-58.50	-3.50	0	0	0	0	0	0	0	0	0	0	0	0	0	0	0	0	0	0	0	0	0
-63.50	-10.50	0	0	0	0	0	0	0	0	0	0	0	0	0	0	0	0	0	0	0	0	0
-74.37	-45.65	0	0	0	0	0	0	0	0	0	0	0	0	0	0	0	0	0	0	0	0	0
-76.50	-8.50	0	0	0	0	0	0	0	0	0	0	0	0	0	0	0	0	0	0	0	0	0
-69.81	-2.46	0	0	0	0	0	0	0	0	0	0	0	0	0	0	0	0	0	0	0	0	0
-75.87	0.08	0	0	0	0	0	0	0	0	0	0	0	0	0	0	0	0	0	0	0	0	0
-75.50	-6.50	0	0	0	0	0	0	0	0	0	0	0	0	0	0	0	0	0	0	0	0	0
-51.50	-26.50	0	0	0	0	0	0	0	0	0	0	0	0	0	0	0	0	0	0	0	0	0
-68.50	2.50	0	0	0	0	0	0	0	0	0	0	0	0	0	0	0	0	0	0	0	0	0
-75.13	-51.55	0	0	0	0	0	0	0	0	0	0	0	0	0	0	0	0	0	0	0	0	0
-39.55	-3.57	0	0	0	1	0	0	0	0	0	0	0	0	0	0	0	0	0	0	0	0	0
-51.50	1.50	0	0	0	0	0	0	0	0	0	0	0	0	0	0	0	0	0	0	0	0	0

Long(x)	Lat(y)	Probu	Cadam	Dhirs	Ecris	Malbi	Mferr	Qcord	Qdich	Qgran	Qmult	Qparv	Sconv	Srufi	Tviri	Tsaya	Tlani	Vcinn	Velli	Vrufa	Vthyr	Vtuca
-50.50	-3.50	0	0	0	0	0	0	0	0	0	0	0	0	0	0	0	0	0	0	0	0	0
-67.50	-40.50	0	0	0	0	0	0	0	0	0	0	0	0	0	0	0	0	0	0	0	0	0
-50.50	-26.50	0	0	0	0	0	0	0	0	0	0	0	0	0	0	0	0	0	0	0	0	0
-72.50	3.50	0	0	0	0	1	0	0	0	0	0	0	0	0	0	0	0	0	0	0	0	0
-75.50	5.50	0	0	0	0	0	0	0	0	0	0	0	0	0	0	0	0	0	0	0	0	0
-50.50	-4.50	0	0	0	0	0	0	0	0	0	0	0	0	0	0	0	0	0	0	0	0	0
-56.48	-27.72	0	0	0	0	0	0	0	0	0	0	0	0	0	1	1	0	0	0	0	0	0
-75.32	-48.51	0	0	0	0	0	0	0	0	0	0	0	0	0	0	0	0	0	0	0	0	0
-59.85	4.23	1	0	0	1	1	0	0	0	0	0	0	0	0	0	0	0	0	0	0	0	0
-59.44	4.55	1	0	0	1	1	0	0	0	0	0	0	0	0	0	0	0	0	0	0	0	0
-58.03	4.11	1	0	0	1	0	0	0	0	0	0	0	0	0	0	0	0	0	0	0	0	0
-58.51	4.51	1	0	0	1	0	0	0	0	0	0	0	0	0	0	0	0	0	0	0	0	0
-59.85	3.66	1	0	0	1	1	0	0	0	0	0	0	0	0	0	0	0	0	0	0	0	0
-59.40	3.45	1	0	0	1	1	0	0	0	0	0	0	0	0	0	0	0	0	0	0	0	0
-58.01	3.98	1	0	0	1	0	0	0	0	0	0	0	0	0	0	0	0	0	0	0	0	0
-58.50	3.50	1	0	0	1	0	0	0	0	0	0	0	0	0	0	0	0	0	0	0	0	0
-61.50	2.50	1	0	0	0	0	0	0	0	0	0	0	0	0	0	0	0	0	0	0	0	0
-60.50	2.50	1	0	0	0	0	0	0	0	0	0	0	0	0	0	0	0	0	0	0	0	0
-54.64	2.18	1	0	0	0	0	0	0	0	0	0	0	0	0	0	0	0	0	0	0	0	0
-54.62	2.72	1	0	0	0	0	0	0	0	0	0	0	0	0	0	0	0	0	0	0	0	0
-54.19	2.49	1	0	0	0	0	0	0	0	0	0	0	0	0	0	0	0	0	0	0	0	0
-53.52	2.14	1	0	0	0	0	0	0	0	0	0	0	0	0	0	0	0	0	0	0	0	0
-53.49	2.63	1	0	0	0	0	0	0	0	0	0	0	0	0	0	0	0	0	0	0	0	0
-61.50	1.50	1	0	0	1	1	0	0	0	0	0	0	0	0	0	0	0	0	0	0	0	0
-60.50	1.50	1	0	0	1	1	0	0	0	0	0	0	0	0	0	0	0	0	0	0	0	0
-46.76	-0.90	1	0	0	0	1	0	0	0	0	0	0	0	0	0	0	0	0	0	1	0	0
-46.51	-1.52	1	0	0	0	1	0	0	0	0	0	0	0	0	0	0	0	0	0	1	0	0
-55.50	-2.50	1	0	0	0	1	0	0	0	1	0	0	1	0	0	0	0	0	0	0	0	0

Long(x)	Lat(y)	Probu	Cadam	Dhirs	Ecris	Malbi	Mferr	Qcord	Qdich	Qgran	Qmult	Qparv	Sconv	Srufi	Tviri	Tsaya	Tlani	Vcinn	Velli	Vrufa	Vthyr	Vtuca
-54.50	-2.50	1	0	0	0	1	0	0	0	1	0	0	1	0	0	0	0	0	0	0	0	0
-43.47	-2.73	1	0	0	0	0	0	0	0	0	0	1	1	0	0	0	0	0	0	0	0	0
-42.61	-2.82	1	0	0	0	0	0	0	0	0	0	1	1	0	0	0	0	0	0	0	0	0
-43.50	-3.50	1	0	0	0	0	0	0	0	1	0	1	0	0	0	0	0	0	0	0	0	0
-42.50	-3.50	1	0	0	0	0	0	0	0	1	0	1	1	0	0	0	0	0	0	0	0	0
-41.50	-3.50	1	0	0	0	1	0	0	0	1	0	1	1	0	0	0	0	0	0	0	0	0
-47.50	-6.50	1	0	0	0	1	1	0	0	1	0	1	0	0	0	0	0	0	0	1	0	0
-45.50	-6.50	1	0	0	0	1	0	0	1	1	1	1	1	0	0	0	0	0	0	0	0	1
-35.51	-6.51	1	0	0	1	1	1	0	0	0	0	0	0	0	0	0	0	0	0	0	0	0
-34.96	-6.79	1	0	0	0	1	1	0	0	0	0	0	0	0	0	0	0	0	0	0	0	0
-47.50	-7.50	1	0	0	0	1	0	0	0	1	1	1	0	0	0	0	0	0	0	1	0	0
-46.50	-7.50	1	0	0	0	1	0	0	0	1	0	1	1	0	0	0	0	0	0	0	0	0
-45.50	-7.50	1	0	0	0	0	0	0	0	1	0	1	1	0	0	0	0	0	0	0	0	0
-44.50	-7.50	1	0	0	0	0	0	0	0	1	0	1	0	0	0	0	0	0	1	0	0	0
-61.50	-8.50	1	0	0	0	1	0	0	0	1	0	1	1	0	0	0	0	0	0	1	0	0
-60.50	-8.50	1	0	0	0	0	0	0	0	0	0	1	0	0	0	0	0	0	0	0	0	0
-55.50	-8.50	1	0	0	0	1	0	0	0	0	0	1	0	0	0	0	0	0	0	0	0	0
-54.50	-8.50	1	0	0	0	1	0	0	0	0	0	1	0	0	0	0	0	0	0	0	0	0
-48.50	-8.50	1	0	0	1	1	1	0	0	1	1	1	1	0	0	0	0	0	0	0	0	0
-47.50	-8.50	1	0	0	0	1	0	0	0	1	0	1	1	0	0	0	0	1	0	0	0	0
-46.50	-8.50	1	0	0	0	1	1	0	0	1	0	1	1	0	0	0	0	0	0	0	0	0
-43.50	-8.50	1	0	0	0	0	0	0	0	0	1	0	0	0	0	0	0	0	1	0	0	0
-61.50	-9.50	1	0	0	0	0	0	0	0	0	0	1	0	0	0	0	0	0	0	0	0	0
-60.50	-9.50	1	0	0	0	0	0	0	0	0	0	1	0	0	0	0	0	0	0	0	0	0
-55.50	-9.50	1	0	0	0	0	0	0	0	1	1	1	1	0	1	0	0	0	0	0	0	1
-47.50	-9.50	1	0	0	0	0	0	0	0	1	0	1	1	0	0	0	0	0	0	1	0	0
-46.50	-9.50	1	0	0	0	1	1	0	0	1	0	1	0	0	0	0	0	0	0	1	0	0
-43.50	-9.50	1	0	0	0	0	0	0	0	0	1	1	0	0	0	0	0	0	1	0	0	0

Long(x)	Lat(y)	Probu	Cadam	Dhirs	Ecris	Malbi	Mferr	Qcord	Qdich	Qgran	Qmult	Qparv	Sconv	Srufi	Tviri	Tsaya	Tlani	Vcinn	Velli	Vrufa	Vthyr	Vtuca
-42.50	-9.50	1	0	0	0	0	0	0	0	0	0	1	0	0	0	0	0	0	0	1	0	0
-41.50	-9.50	1	0	1	0	0	0	0	0	0	0	0	0	0	0	0	0	0	0	1	0	0
-36.50	-9.50	1	0	0	0	1	1	0	0	0	0	0	0	0	0	1	0	0	0	0	1	0
-65.18	-10.51	1	0	0	0	0	0	0	0	1	1	0	1	0	0	0	0	0	0	1	0	0
-65.68	-10.49	1	0	0	0	0	0	0	0	1	1	0	1	0	0	0	0	0	0	1	0	0
-53.50	-10.50	1	1	1	0	1	1	1	1	1	1	1	1	0	0	0	1	1	1	1	1	1
-52.50	-10.50	1	1	1	0	1	1	1	1	1	1	1	1	0	0	0	1	1	1	1	1	1
-49.50	-10.50	1	0	0	0	0	0	0	1	1	1	1	0	0	0	0	0	0	0	1	0	0
-48.50	-10.50	1	0	0	1	1	1	0	0	1	1	1	1	0	0	0	0	1	1	1	0	0
-47.50	-10.50	1	0	0	1	1	1	0	0	1	1	1	1	0	0	0	0	1	1	1	0	0
-46.50	-10.50	1	0	0	0	1	1	0	0	1	1	1	1	0	0	0	0	1	1	1	0	0
-45.50	-10.50	1	0	0	0	1	0	0	1	1	0	1	1	0	0	0	0	0	0	0	0	0
-37.51	-10.49	1	0	0	0	1	1	0	0	0	0	0	0	0	0	0	0	0	0	0	1	0
-36.64	-10.30	1	0	0	0	1	1	0	0	0	0	0	0	0	0	0	0	0	0	0	0	0
-53.50	-11.50	1	1	1	0	1	1	1	1	1	1	1	1	0	0	0	1	1	1	1	1	1
-52.50	-11.50	1	1	1	0	1	1	1	1	1	1	1	1	0	0	0	1	1	1	1	1	1
-51.50	-11.50	1	0	0	0	0	0	0	1	1	1	1	1	0	0	0	0	1	0	1	0	0
-50.50	-11.50	1	0	0	0	0	0	0	1	1	1	1	1	0	0	0	0	1	0	1	0	0
-49.50	-11.50	1	0	0	0	1	0	0	0	0	1	1	1	0	0	0	0	0	0	1	0	0
-47.50	-11.50	1	0	0	0	1	1	0	0	1	1	1	1	0	0	0	0	1	1	1	1	0
-46.50	-11.50	1	0	0	0	1	1	0	0	1	1	1	1	0	0	0	0	1	1	1	0	0
-45.50	-11.50	1	0	0	0	1	0	0	1	1	1	1	1	0	0	0	0	0	0	1	1	1
-41.50	-11.50	1	0	1	0	1	0	1	1	0	0	0	0	0	0	0	0	0	0	0	1	1
-40.50	-11.50	1	0	1	0	1	1	1	1	0	0	0	0	0	0	0	0	0	0	0	1	0
-37.68	-11.43	1	0	0	0	1	1	0	0	0	0	1	0	0	0	0	0	0	0	0	0	0
-60.50	-12.50	1	0	0	0	0	0	0	0	1	1	1	1	0	0	0	0	1	0	1	0	0
-51.50	-12.50	1	0	0	0	1	0	0	0	1	1	1	1	0	0	0	0	0	0	1	0	0
-46.50	-12.50	1	1	0	1	1	1	0	0	1	1	1	1	0	0	0	0	0	1	1	1	0

Long(x)	Lat(y)	Probu	Cadam	Dhirs	Ecris	Malbi	Mferr	Qcord	Qdich	Qgran	Qmult	Qparv	Sconv	Srufi	Tviri	Tsaya	Tlani	Vcinn	Velli	Vrufa	Vthyr	Vtuca
-42.50	-12.50	1	0	0	0	1	0	0	1	1	0	1	1	0	0	0	0	0	1	0	0	1
-41.50	-12.50	1	0	0	1	1	0	1	1	1	0	1	1	0	0	1	0	0	0	0	1	1
-38.52	-12.44	1	0	0	1	1	1	0	0	0	0	0	0	0	0	0	0	0	0	0	1	0
-37.87	-12.18	1	0	0	1	1	0	0	0	0	0	1	0	0	0	0	0	0	0	0	0	0
-56.50	-13.50	1	0	0	1	1	1	0	1	0	1	0	1	0	0	0	0	1	0	0	0	1
-51.50	-13.50	1	0	0	0	1	0	0	0	1	1	1	1	0	1	0	0	0	0	1	0	0
-50.50	-13.50	1	0	0	0	0	0	0	0	1	1	1	0	0	0	0	0	1	0	1	0	0
-49.50	-13.50	1	0	0	0	0	0	0	0	1	0	1	1	0	1	1	0	1	0	1	0	0
-48.50	-13.50	1	0	0	0	1	1	0	1	1	1	1	1	0	1	1	0	0	1	1	0	1
-47.50	-13.50	1	1	0	1	1	1	0	1	1	1	1	1	0	1	1	0	1	1	1	1	1
-46.50	-13.50	1	1	0	0	1	1	0	1	1	1	1	1	0	0	1	0	1	1	1	1	1
-45.50	-13.50	1	0	0	0	1	1	0	0	1	0	1	1	0	0	0	0	0	1	1	1	0
-44.50	-13.50	1	0	0	0	0	0	0	0	1	1	1	0	0	0	0	0	1	0	1	0	0
-42.50	-13.50	1	0	1	0	1	1	1	1	1	1	1	1	0	0	0	0	0	1	1	1	1
-41.50	-13.50	1	0	1	1	1	1	1	1	1	0	1	1	0	1	0	0	0	1	0	1	1
-38.90	-13.26	1	0	0	1	1	0	0	0	0	0	0	0	0	0	0	0	0	0	0	0	0
-56.50	-14.50	1	0	0	1	1	1	0	0	1	1	1	1	0	0	0	0	1	0	1	0	0
-52.50	-14.50	1	0	0	0	1	0	0	0	1	1	1	1	0	0	0	0	1	0	1	0	0
-51.50	-14.50	1	0	0	0	1	0	0	0	0	1	1	1	0	1	0	0	0	0	1	0	0
-50.50	-14.50	1	0	0	0	1	0	0	0	1	0	1	1	0	0	0	0	0	0	0	0	0
-49.50	-14.50	1	0	0	0	1	1	0	0	1	1	1	1	0	1	1	0	1	0	1	0	0
-48.50	-14.50	1	1	0	0	1	1	0	1	1	1	1	1	0	1	1	0	0	0	1	0	1
-47.50	-14.50	1	1	0	1	1	1	0	1	1	1	1	1	0	1	1	0	1	1	1	1	1
-46.50	-14.50	1	0	0	1	1	1	0	1	1	1	1	1	0	0	0	0	1	1	1	1	1
-45.50	-14.50	1	0	0	1	1	0	0	0	1	1	1	0	0	0	0	0	1	1	1	0	1
-44.50	-14.50	1	0	0	0	1	0	0	0	1	1	1	1	0	0	0	0	1	1	0	1	0
-42.50	-14.50	1	0	0	1	1	1	1	1	1	1	1	0	0	0	0	0	0	1	0	1	1
-54.50	-15.50	1	0	0	0	1	1	0	0	1	0	1	1	0	0	0	0	0	1	1	0	0

Long(x)	Lat(y)	Probu	Cadam	Dhirs	Ecris	Malbi	Mferr	Qcord	Qdich	Qgran	Qmult	Qparv	Sconv	Srufi	Tviri	Tsaya	Tlani	Vcinn	Velli	Vrufa	Vthyr	Vtuca
-53.50	-15.50	1	0	0	0	0	0	0	0	1	0	1	1	0	0	0	0	0	1	1	0	0
-50.50	-15.50	1	0	0	0	1	1	0	0	1	1	1	1	0	0	0	0	0	1	1	0	1
-49.50	-15.50	1	1	0	1	1	1	1	1	1	1	1	1	0	0	1	0	0	1	1	1	1
-48.50	-15.50	1	1	0	1	1	1	1	1	1	1	1	1	0	0	0	0	1	1	1	1	0
-47.50	-15.50	1	1	0	1	0	1	0	1	1	1	1	1	0	0	1	0	1	0	1	1	1
-46.50	-15.50	1	1	0	1	1	1	0	1	1	1	1	1	0	0	0	0	0	1	1	1	1
-45.50	-15.50	1	1	0	1	1	1	1	0	1	1	1	1	0	0	0	0	0	1	1	1	1
-42.50	-15.50	1	0	1	0	1	1	1	1	0	0	1	1	0	0	0	0	0	1	1	1	1
-52.50	-16.50	1	1	0	0	1	1	0	1	1	1	1	1	0	0	1	0	1	1	1	1	1
-51.50	-16.50	1	1	0	0	1	1	0	1	1	1	1	1	0	0	0	0	1	1	1	0	1
-50.50	-16.50	1	1	0	0	1	1	0	0	1	1	1	1	0	0	0	0	0	1	1	0	1
-49.50	-16.50	1	1	0	0	1	1	0	1	1	1	1	1	0	1	1	0	1	0	1	0	1
-48.50	-16.50	1	1	0	0	1	1	0	1	1	1	1	1	0	0	1	0	1	1	1	1	1
-47.50	-16.50	1	1	0	1	1	1	1	1	1	1	1	0	0	0	0	0	1	1	1	1	1
-46.50	-16.50	1	0	1	0	1	1	1	1	1	1	1	1	0	0	0	0	0	1	1	0	0
-45.50	-16.50	1	0	1	0	1	0	1	0	1	1	1	1	0	0	0	0	1	0	1	1	0
-43.50	-16.50	1	1	1	1	1	1	1	1	1	0	1	0	0	0	0	0	0	1	1	1	1
-42.50	-16.50	1	1	1	1	1	1	1	1	1	0	1	0	0	0	0	0	0	1	1	1	1
-39.53	-16.48	1	0	0	0	1	0	1	1	0	0	0	0	0	0	0	0	0	0	0	1	1
-38.97	-16.08	1	0	0	0	1	0	1	1	0	0	0	0	0	0	0	0	0	0	0	1	0
-53.50	-17.50	1	1	0	1	1	1	0	0	1	1	1	0	0	0	0	0	0	0	0	0	1
-52.50	-17.50	1	1	0	0	1	1	0	1	1	1	1	1	0	0	0	0	1	0	1	0	1
-51.50	-17.50	1	1	0	0	1	1	0	1	1	1	1	1	0	0	0	0	1	1	1	0	1
-50.50	-17.50	1	1	0	0	1	1	0	1	1	1	1	1	0	0	0	0	0	1	1	0	1
-48.50	-17.50	1	1	0	0	1	1	1	1	1	1	1	1	0	0	0	0	0	1	1	0	1
-47.50	-17.50	1	1	0	0	1	1	1	1	1	1	1	1	0	0	0	0	1	1	1	0	1
-46.50	-17.50	1	0	0	0	1	1	1	1	1	1	1	1	0	0	0	0	0	1	1	1	1
-45.50	-17.50	1	0	0	0	1	0	1	0	1	1	1	1	0	0	0	0	0	1	1	1	1

Long(x)	Lat(y)	Probu	Cadam	Dhirs	Ecris	Malbi	Mferr	Qcord	Qdich	Qgran	Qmult	Qparv	Sconv	Srufi	Tviri	Tsaya	Tlani	Vcinn	Velli	Vrufa	Vthyr	Vtuca
-43.50	-17.50	1	1	1	1	1	1	1	1	1	1	1	1	0	0	0	1	1	1	1	1	1
-42.50	-17.50	1	1	0	1	0	1	1	1	0	0	1	0	0	0	0	0	0	1	0	1	1
-53.50	-18.50	1	1	0	0	1	1	0	0	1	1	1	1	0	0	0	0	1	1	1	1	0
-52.50	-18.50	1	1	0	0	1	1	0	0	1	1	1	1	0	0	0	0	1	0	1	0	1
-51.50	-18.50	1	1	0	0	1	0	0	1	1	1	1	1	0	0	1	0	1	1	1	0	1
-48.50	-18.50	1	1	0	1	1	1	1	1	1	1	1	1	0	0	0	0	1	1	1	0	1
-47.50	-18.50	1	1	0	1	1	1	0	1	1	1	1	1	0	0	0	0	1	1	1	1	1
-45.50	-18.50	1	1	0	0	1	1	1	0	1	1	1	1	0	0	0	1	0	1	1	1	1
-44.50	-18.50	1	1	1	0	1	0	1	1	1	1	1	1	0	1	0	0	1	1	1	1	0
-43.50	-18.50	1	1	1	0	1	1	1	1	1	1	1	1	0	1	0	1	1	1	1	1	1
-42.50	-18.50	1	1	0	0	0	1	1	1	1	0	1	0	0	0	1	0	0	1	0	1	0
-56.50	-19.50	1	0	0	0	0	0	0	0	1	0	1	0	0	0	0	0	1	0	0	0	0
-55.50	-19.50	1	1	0	0	1	1	0	1	1	1	1	0	0	0	0	0	1	0	0	0	0
-48.50	-19.50	1	1	0	0	1	1	0	1	1	1	1	1	0	0	0	0	1	1	1	0	1
-47.50	-19.50	1	1	0	1	1	1	0	1	1	1	1	1	0	0	0	0	1	0	1	0	1
-44.50	-19.50	1	1	1	1	1	1	1	1	1	1	1	1	1	1	0	1	0	1	1	1	1
-43.50	-19.50	1	1	1	1	1	1	1	1	1	1	1	1	1	0	0	1	1	1	1	1	1
-40.51	-19.49	1	0	0	0	1	0	0	0	0	0	0	0	0	0	1	0	0	0	0	0	0
-47.50	-20.50	1	1	0	1	1	1	0	1	1	1	1	1	0	0	0	0	1	1	1	0	1
-46.50	-20.50	1	1	0	1	1	1	1	1	1	1	1	1	1	1	0	0	1	1	0	1	1
-45.50	-20.50	1	0	0	0	1	1	1	0	1	1	1	0	0	0	0	0	1	0	0	1	1
-44.50	-20.50	1	1	1	1	1	1	1	1	1	1	1	0	0	0	1	1	0	1	0	1	1
-43.50	-20.50	1	1	1	1	1	1	1	1	1	1	0	0	0	0	1	1	1	0	0	1	1
-40.67	-20.40	1	0	0	0	1	0	0	0	0	0	0	0	0	0	1	0	0	0	0	0	0
-47.50	-21.50	1	1	0	1	1	0	0	1	1	1	1	0	0	0	1	0	1	1	0	0	1
-46.50	-21.50	1	0	0	0	1	1	1	0	1	1	1	0	0	0	1	0	0	0	0	1	1
-45.50	-21.50	1	0	1	0	1	1	1	0	1	1	0	0	0	0	0	0	0	0	0	1	1
-44.50	-21.50	1	1	1	1	1	1	1	1	1	1	1	0	0	0	1	0	1	0	0	1	1

Long(x)	Lat(y)	Probu	Cadam	Dhirs	Ecris	Malbi	Mferr	Qcord	Qdich	Qgran	Qmult	Qparv	Sconv	Srufi	Tviri	Tsaya	Tlani	Vcinn	Velli	Vrufa	Vthyr	Vtuca
-43.50	-21.50	1	1	0	1	1	0	1	1	1	0	0	0	0	0	1	0	0	0	0	0	1
-42.50	-21.50	1	0	1	0	1	1	0	0	0	0	0	0	0	0	0	0	0	0	0	0	1
-41.50	-21.50	1	0	0	0	1	1	0	0	0	0	0	0	0	0	0	0	0	0	0	0	0
-46.50	-22.50	1	1	0	0	1	0	1	1	1	1	1	0	0	0	1	0	1	0	0	0	1
-44.50	-22.49	1	0	0	0	1	0	1	0	0	1	0	0	1	1	1	0	0	0	0	0	1
-42.50	-22.48	1	0	1	0	1	1	0	1	0	1	0	0	1	1	1	0	0	0	0	0	0
-41.67	-22.17	1	0	0	0	1	1	0	0	0	0	0	0	0	0	0	0	0	0	0	0	0

Table S4 Performance of classification model between different algorithms for presences and absences.

	Presence			Absence		
Algorithms	Precision	Recall	F1-score	Precision	Recall	F1-score
RandomForest	0.91	0.90	0.90	0.91	0.91	0.91
CatBoost	0.90	0.87	0.89	0.88	0.91	0.89
XGBoost	0.90	0.90	0.90	0.91	0.91	0.91

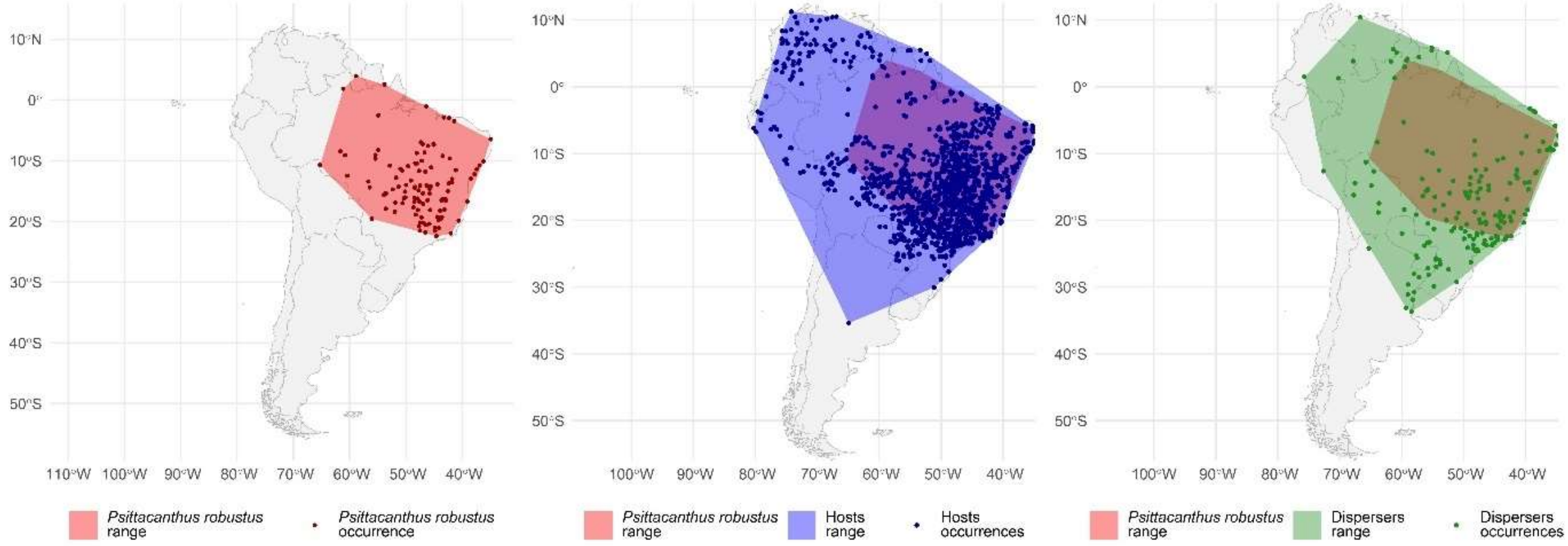

Fig. S1 Range and occurrences points of *Psittacanthus robustus*, combined hosts and combined dispersers in the South America.

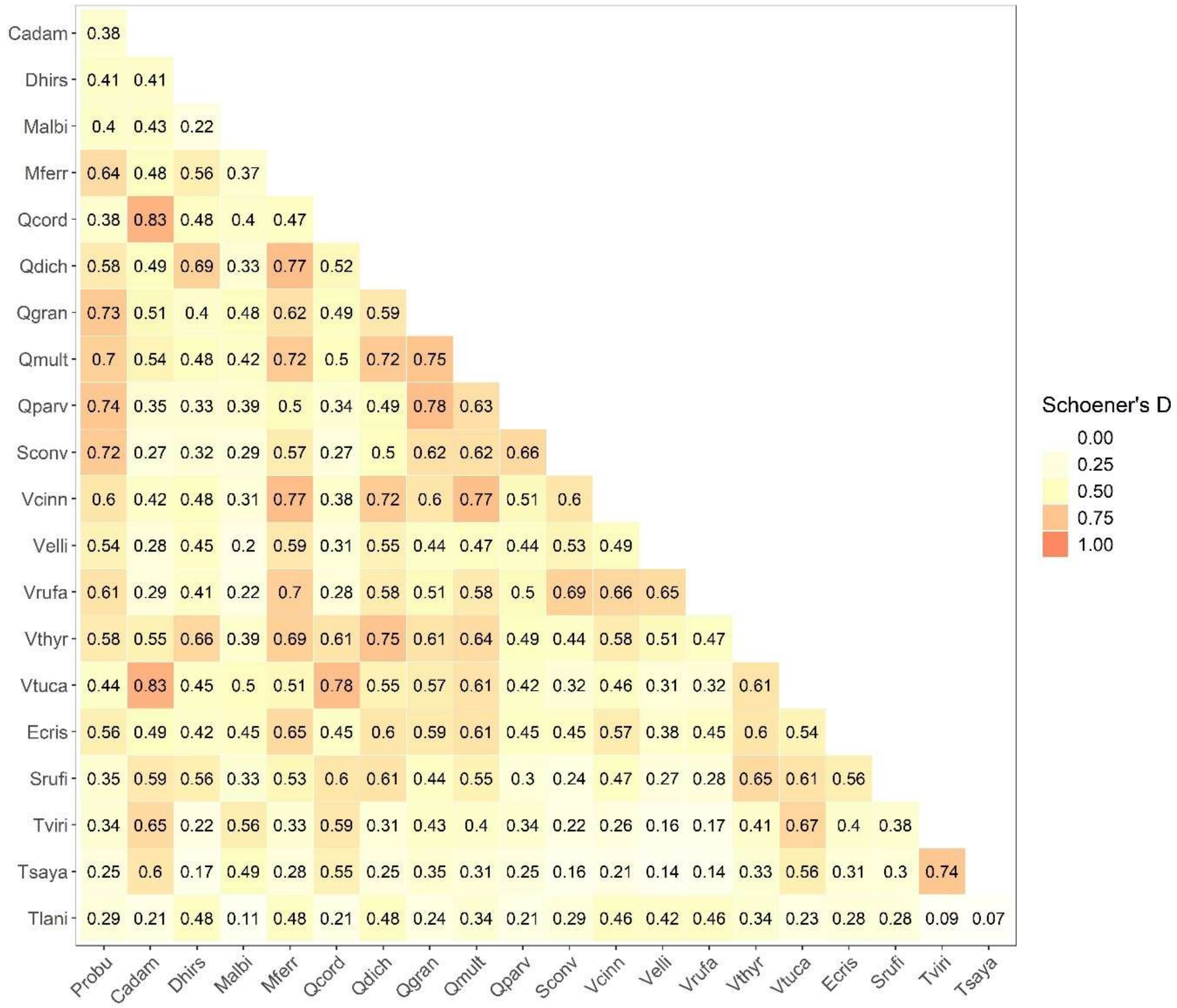

Fig. S2 Observed values of Schoener's D index for climatic niche overlap between each pair of species. The significance of the niche equivalence test is shown below the D-index values (* = p <0.05). Abbreviations: Probu= *Psittacanthus robustus*; Cadam= *Campomanesia adamantium*; Dhirs= *Diplusodon hirsutus*; Qcord= *Qualea cordata*; Qdich= *Qualea dichotoma*; Qgran= *Qualea grandiflora*; Qmult= *Qualea multiflora*, Qparv= *Qualea parviflora*; Malbi= *Miconia albicans*; Mferr= *Miconia ferruginata*; Sconv= *Salvertia convallariodora*; Tlani= *Trembleya laniflora*; Vcinn= *Vochysia cinnamomea*; Velli= *Vochysia elliptica*; Vrufa= *Vochysia rufa*; Vthyr= *Vochysia thyrsoidea*; Vtuca= *Vochysia tucanorum*; Ecris= *Elaenia cristata*; Srufi= *Schistochlamys ruficapillus*; Tviri= *Tersina viridis viridis*; Tsaya= *Thraupis sayaca sayaca*.

Text S1. Procedures established to perform the literature search used to identify host plant and seed-dispersing bird species associated with *Psittacanthus robustus*.

We conducted a comprehensive systematic search of the literature to compile records of host plant and seed-dispersing bird species associated with *Psittacanthus robustus*. Searches were performed in June 2026 using the following bibliographic databases: Web of Science, Scopus, SciELO, Biological Abstracts, and Google Scholar. Additional records were identified through backward citation tracking of the reference lists of relevant publications.

Searches followed Boolean logic (AND/OR) and included combinations of the scientific name of the mistletoe with terms related to host associations and seed dispersal. The primary search string in English was: "*Psittacanthus robustus*" AND (host* OR "host plant*" OR parasiti* OR dispers* OR "seed dispers*" OR bird* OR avian). Equivalent search terms were also applied in Portuguese and Spanish to maximize the retrieval of studies published in different languages. No restrictions were imposed regarding publication year.

We included only studies reporting direct observations or documented records of host plant species or seed-dispersing birds associated with *P. robustus*. Taxonomic names were standardized according to their currently accepted nomenclature (CTFB 2026; Flora e Funga do Brasil 2026), and only records identified to the species level (binomial scientific names) were included in the final dataset.

References

CTFB-Catálogo Taxonômico da Fauna do Brasil 2026. Brazilian Zoology Group. Available at <https://fauna.jbrj.gov.br/fauna/>. Access on: 14 Jun 2026

Flora e Funga do Brasil. Jardim Botânico do Rio de Janeiro. Available at <http://floradobrasil.jbrj.gov.br/ >. Access on: 14 Jun 2026
